# The messenger matters: Behavioral responses to sex education in a cluster randomized trial

**DOI:** 10.1093/pnasnexus/pgag183

**Published:** 2026-05-22

**Authors:** Noam Angrist, Gabriel Anabwani

**Affiliations:** Blavatnik School of Government, Oxford University, 120 Walton St, Oxford OX2 6GG, United Kingdom; Youth Impact, Plot 6789, Seboko. Ext. 21, Broadhurst, Gaborone, Botswana; Botswana-Baylor Children’s Clinical Centre of Excellence, Plot 1836, Hospital Way, Gaborone, Botswana

**Keywords:** sex education, education, government policy, information, persuasion

## Abstract

This article estimates the effect of sex education in a large-scale cluster randomized trial covering a third of Botswana that varied both the message and the messenger. Educational messages delivered by near-peers led to a statistically significant 40% reduction in adolescent pregnancy incidence, whereas the same messages delivered by government teachers showed no detectable effect. While both types of messengers successfully change students’ beliefs, students appear to be persuaded by near-peers to change their behavior but are not persuaded by teachers. These results demonstrate the first-order role messengers can play in influencing behavior and motivate greater use of near-peer messengers to deliver sex education at scale.

Significance statement We evaluate multiple scalable models to deliver sex education in a large randomized trial in Botswana. We test the effectiveness of near-peers relative to teacher messengers. While both messengers change student beliefs, we find near-peers are effective at changing behavior, while teachers are not. These results motivate greater use of near-peer models, such as widespread government National Service Programs, to deliver sex education at scale. Our results have broad implications for the effectiveness of government programs—the messenger delivering a given message is of critical importance.

## Introduction

Many governments include sex education in school curricula in an effort to curb risky sex. The stakes are high: the United Nations estimates that as of 2022, 1.3 million HIV infections still occur worldwide every year (1). Effective sex education is of particular importance in sub-Saharan Africa, which accounts for 70% of new HIV infections and where nearly 1 in 10 adults has HIV (2). Yet the question of how sex education can be delivered most effectively remains unclear. While a growing body of evidence exists on sex education messages that can reduce risky sex, less is known about the messenger.

Understanding the importance of the messenger is critical. The typical messengers for sex education are teachers, but it is uncertain if teachers are the ideal messenger. Teachers are respected authority figures and might be most effective at changing students’ beliefs. On the other hand, students might ignore or rebel against authority and not change their behavior. Alternative messengers, such as near-peers, who are young and aspirational figures, might be most persuasive.^a^ Many countries have national service programs which deploy near-peers in schools and could deliver sex education at scale. Indeed, over 15 governments in sub-Saharan Africa have such programs, investing over $240 million per year (3).

However, identifying messenger effects is challenging. Observational studies suggest sex education delivered by teachers has yielded limited success (4), yet such studies rarely compare multiple messengers since in the status quo teachers are almost always the default messenger. Moreover, experimental studies of information interventions often bundle the messenger and message together, rendering messenger effects difficult to isolate (5–10).

In this article, we estimate sex education effects, isolating the effect of the messenger from the message in a three-armed clustered randomized trial. One arm is a control group, a second is delivery of sex education by teachers, and the third is delivery of the same sex education message by near-peers. Our study takes place in Botswana and was conducted at scale with the government, covering 42,000 students and 343 schools across a third of the country. Data were collected on risky sexual behavior, measured by teenage pregnancy, with a 99% response rate 12–15 months later. This teenage pregnancy outcome, which has an unusually high long-term tracking success rate and which captures a highly consequential outcome, enables estimation on objective behavioral responses and an assessment of whether students were persuaded to change their behavior. Moreover, data were collected on compliance to see which messengers delivered the message as expected, and beliefs, to explore which messengers were believed by students. Together, these comprehensive indicators enable both evaluation of messenger effectiveness as well as exploration of mechanisms along various messenger channels.

In terms of messenger characteristics, near-peer messengers are 20 years younger than teachers, on average, rarely have formal teaching experience, and are role model figures.^b^ The sex education message provided information that younger partners are less likely to have HIV than older partners and are a safer sex option—a message shown to be highly effective and reduce pregnancy by 28% in a randomized trial in Kenya (6). We hold the message consistent with a message already shown to be effective for two reasons. First, this enables us to assess replicability of a similar sex education message across diverse settings—an important question in itself. Second, if the message tested does not change behavior in the first place then we cannot assess the marginal effect of the messenger. By building on prior effective messaging, we can provide novel insight on messenger effects.

We contribute new evidence on messenger effects in the context of sex education. A growing body of evidence shows that sex education messages can reduce risky sex but that the type of information matters (6, 11–15).^c^ For example, messages about safe sex options are more effective at reducing risky sex than abstinence-only messages. However, very few randomized studies to date exist on the effects of the messenger (16). This study builds on related contributions of messenger effects in the broader persuasion literature, which includes messenger effects for public health vaccination campaigns as well as voting campaigns (17–20). Evaluating the most typical messengers of sex education at scale—government teachers—relative to alternative messengers, such as near-peers, is a key contribution of this study. The importance of the messenger can help reconcile why despite field experiments in Africa which have found large responses to information (6, 21, 22)—and which often use messengers with more similar characteristics to near-peers—risk responses are much smaller at the population-level (4) where teachers are the default messenger. This study is also one of the largest sex education randomized trials conducted to date, ensuring statistical power to estimate effects on consequential rare events, such as teen pregnancy, and contributing to a growing science of scale literature (23).

## Intervention and study design

### Sex-education message

A study in Kenya found that revealing that older partners are significantly more likely to have HIV than younger partners led to a large reduction in teen pregnancy of 28% (6). We deliver a similar sex education message in Botswana. Similar to Kenya, HIV rates increase dramatically with age in Botswana. Figure S1 shows the HIV rate by age and gender in Botswana (24). This figure illustrates two important facts. First, HIV rates increase substantially with age. Second, young girls are likely getting infected by older partners, since their infection rate increases substantially faster than their male peers.

Despite the dire risks older partners pose, intergenerational relationships are common. According to a study by PSI, 12 to 25% of young women in sub-Saharan Africa had partners ten or more years their senior (25). The pervasiveness of relationships with older partners is common in many settings (26) and is driven by a few factors. Among other reasons, girls often misperceive these older men as having lower, rather than higher, rates of HIV, and often enter these relationships unaware of the severe risks.

The sex education message studied reveals the relative HIV risk of older versus younger male sexual partners. This makes the previously underestimated cost of older partners known and salient, and also makes clear that dating age-mates is a relatively safer sex option.

This intervention contrasts with the typical sex education message—abstinence—which emphasizes risk avoidance until marriage. Figure S2 shows an example of a typical sign at a school which emphasizes abstinence. Abstinence messages fail to provide a realistic safe sex option to youth who will not abstain. If sexual behavior is more likely to change on the relative intensive margin (type of sex) rather than the absolute extensive margin (sex or no sex), providing safe sex alternatives, such as revealing younger partners are lower risk than older partners, will be more effective.

The message was delivered through a brief 1-h class in government schools which included revealing a version of the HIV risk graph in Fig. S1, a short video of a young girl overcoming an older partner’s advance, and a discussion. This near-identical curriculum and message from Kenya to Botswana enables an assessment of external validity of the message across contexts.

While treatment options for HIV have expanded substantially in recent years, helping control the spread of HIV, high rates of HIV continue to persist (27). Moreover, HIV treatment options do not prevent teenage pregnancy. Thus, cost-effective risky sex prevention approaches, such as sex education, remain important (28).

### Messengers: near-peers and teachers

We randomize near-peers and teachers to deliver the same sex education message. The control group consists of teachers delivering a status-quo curriculum. This design enables us to capture the effect of the sex education message and the relative effect of the messenger when delivering the sex education message.

In the near-peer arm, young and aspirational figures were selected from a competitive application pool to deliver the sex education message. Near-peers is a common term in the public health literature. While peers are typically the same age as a given recipient of information and often in the same social network, near-peers are typically similar in age yet slightly older and are aspirational role model figures.

In the teacher arm, one teacher was nominated per school to receive training. The criteria for selection was to ensure the teacher was a guidance and counseling teacher. This ensured delivery of sex education was in their job description to increase the likelihood of delivery. It further enables comparison with the control group where guidance and counseling teachers are expected to deliver the status-quo curriculum.

Each messenger represents a bundle of characteristics. Appendix Table S1 describes messenger characteristics such as gender, degree and teaching experience. Near-peer educators are substantially younger than teachers, on average, by 20 years. Gender composition is similar: 88% of near-peers are female relative to 80% female for teachers. In terms of teaching experience, near-peers have limited experience, relative to teachers who have an average of 20 years of teaching experience. Along this bundle of characteristics for which we have data, near-peers messengers appear significantly more proximate to students who receive the sex education message than teachers. Near-peers are also more likely to obtain a university bachelor’s degree, typically associated with being more aspirational figures.

Near-peers were trained for five days on the intervention by a local NGO, Youth Impact, and two professors from the Department of Primary Education at the University of Botswana. Near-peers were paid a small daily stipend during the program. Once at the school, one near-peer was allocated to each classroom ranging from 31 to 40 students on average. All classrooms in a school were reached simultaneously during study hour. All schools randomized to receive treatment were reached within 40 working days. Random monitoring visits were conducted by the Ministry of Education, and the Botswana Baylor Children’s Clinical Center of Excellence, the largest HIV clinic in the country, and NGO staff.

Teachers received training hosted by the Ministry of Education in each of the four regions in the sample. An official of the Ministry of Education opened each training. The training was delivered by the same head facilitator from the NGO and professors from the University of Botswana who trained the near-peer arm on the same five segments to provide a near-identical intervention. All teachers were requested to sign commitment cards indicating when they planned to implement the intervention within the term. This served as a commitment device. All teachers were sent reminder text messages to implement the intervention and were asked to give students written reflections to be turned into the regional Ministry of Education authorities for verification of implementation. Random monitoring visits were conducted by a monitoring team in both arms.

Both teachers and near-peers were expected to deliver the intervention during an established study hour where the curriculum often rotates subjects and includes topics such as comprehensive sexuality education. This ensured similar delivery protocols for both treatments and integration with the existing government system.

It is not clear ex ante if teachers or near-peers will be more effective. Near-peers might be less effective at changing beliefs due to lack of experience and authority. Therefore, recipient behavior might not change. On the other hand, near-peers might be more reliable implementers as well as proximate and credible messengers. This might result in greater shifts in beliefs and corresponding sexual behavior change. Alternatively, teachers and near-peers might be similarly effective at changing beliefs but differentially effective at changing behavior. This article sheds light on messenger effects and mechanisms in the context of sex education.

### Context and experimental design

The study took place from 2014 to 2015 in Botswana in a third of the country. The study is a three-armed randomized trial conducted in government schools. A total of 347 schools were included in the initial sampling frame across three grade levels: grade 6 in primary schools and grade 8 and grade 9 in junior secondary schools. This encompasses the universe of all schools in each region according to administrative records from the Ministry of Education. Schools not listed on official government lists were not included in the randomization.

Of the 347 schools in the original sample, 115 schools were selected for sex education messaging by near-peers, 116 schools by teachers, and 116 schools selected for the control group. Of the 347 schools in the initial sample, 343 schools were included in the final sample (113 near-peer/114 teacher/116 control). Four schools were excluded: two were excluded as they did not have grade 6 students, another was excluded as it was a private school, and a final school was not able to be found. The final sample includes 273 primary schools and 70 junior schools, with roughly 42,000 youth enrolled, of which about 22,000 are young girls, who are the primary focus of the analysis. The randomization was conducted based on the 343 schools in this final sample, stratified by region and school level (primary and junior). Figure S3 shows a map of all schools in the study.

Table S2 presents summary statistics and balance tests across survey data as well as administrative data on test scores, dropout, and pregnancy. We find overall balance, with only a few variables showing small differences, and an overall omnibus joint F-test showing balance. To account for potential differences among indicators and to enhance statistical power we control for a vector of baseline covariates. These variables include: male and female enrollment, gender composition, average age, baseline dropouts, self-reported rates of pregnancy, the percent of girls in the class reported to be pregnant, the percent of girls in the class reported to have older boyfriends, and a baseline literacy index.

Figure S4 provides a study timeline. The baseline survey commenced in August 2014, followed by implementation which extended through November 2014. The endline survey commenced 12 months later in August 2015 and was complete by the end of December 2015. Follow-up occurred within schools at the next grade level assuming standard progression. We achieved nearly 100% follow-up, with all 343 schools and grade levels in the baseline surveyed 12 months later except one grade in one school.

The study occurred in four regions, representing over a third of the country, and included both rural and urban settings. One region includes the capital and is highly urbanized. The other three regions are largely rural. The intervention was conducted in late primary school and lower secondary schools with students ranging in age from 12 to 16. This age range covers adolescents around the age of sexual debut. While exact data on age of sexual debut is inconsistent, the sum of the evidence suggests the average first sexual encounter occurs between ages 15–19. While only 4% of youth in Botswana younger than 15 have had sex, over 60% of youth 19 or older are sexually active (24, 29). During this phase of adolescence, sex education can influence a large margin of students since sexual relationships are just starting to be formed.

Moreover, most students in this age range are easy to reach since they are in school. The primary net enrollment rate in Botswana is 90% (30). The transition rate from primary to junior school is 97% (31). However, there is a sharp drop-off between lower and upper secondary school of over 30% of students (31). Thus, school-based interventions that intervene before this large drop-off can reach most students before negative outcomes occur.

Each school in Botswana has a dedicated Guidance and Counseling teacher who is expected to deliver sex education over the course of the year. In practice, while every school has dedicated teachers and time to deliver sex education, over 80% of students in Botswana have never heard of the sex education curriculum (29). This presents an opportunity to activate delivery of sex education messaging in Botswana and introduce safe sex strategies. To this end, we examine the effect of a sex education message shown to work in a prior context and explore differential effects by messenger when delivered by near-peers and teachers.

## Results

### Beliefs

We analyze beliefs among adolescent girls immediately after the intervention. The beliefs most likely to affect sexual behavior by endline are closest to the period shortly after the intervention since it takes 6 to 9 months for observable pregnancy to occur. A supplemental analysis of immediate effects on beliefs includes analysis of beliefs 12 months later for two reasons. First, if trends a year later exhibit similar trends to those immediately after the intervention, this increases our confidence that these trends hold in the following months after the intervention. Second, persistence of beliefs increases the likelihood of long-run impact.

**Fig. 1.**
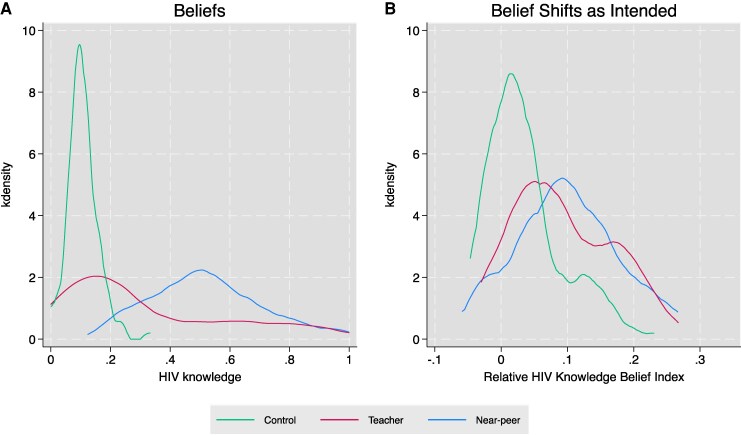
Student beliefs. This figure presents beliefs by treatment arm in both panels. We show treatment effects on beliefs measured by HIV knowledge after the intervention. HIV knowledge is defined as knowing 40-y-old men are most likely to have HIV (among various age group categories students could select). In Panel (A), the figure shows distributions based on the percentage of students who answer this question correctly. In Panel (B), we also present treatment effects on how beliefs shift as intended, understanding relative risks not just absolute risks. The index of beliefs captures the degree to which students think older partners have higher HIV than younger partners. The index is a linear subtraction of correctly identifying 40-y-old men as mostly to have HIV minus incorrectly identifying 10- to 19-y-old men as most likely to have HIV. Belief outcomes include all girls.

Figure 1A illustrates beliefs graphically for HIV knowledge transferred shortly after the intervention. These beliefs relate to a specific belief indicator: knowledge of which partner age-group is most likely to have HIV (eg men aged 40–44). Table 1 also shows shifts in beliefs and includes both beliefs for HIV-specific risks by partner age group (eg 40-y-old men) as well as more general risk perceptions by broader age categories (eg older men). Figure 1A shows large shifts in correct risk beliefs right after the intervention in the near-peer arm in columns 1 and 2 with a 44.2 (P<0.001) and 33.0 (P<0.001) percentage point increase in HIV age category and general HIV risk knowledge. There is a large although smaller shock to beliefs in the teacher arm of 17.4 (P<0.001) and 12.0 (P=0.002) percentage points. Twelve months later about a quarter of all belief outcomes are retained among both near-peers and teachers as shown in columns 3 and 4.

**Table 1.** Student beliefs

	Beliefs postintervention		Beliefs after 12 months	
	(1)	(2)	(3)	(4)
	HIV belief	Risk belief	HIV belief	Risk belief
Near-peer arm	0.442	0.330	0.088	0.086
	(0.017)	(0.014)	(0.008)	(0.013)
	[0.409, 0.476]	[0.303, 0.357]	[0.073, 0.102]	[0.059, 0.112]
	{0.000}	{0.000}	{0.000}	{0.000}
Teacher arm	0.174	0.120	0.042	0.031
	(0.038)	(0.038)	(0.009)	(0.016)
	[0.100, 0.249]	[0.045, 0.194]	[0.024, 0.059]	[−0.000, 0.063]
	{0.000}	{0.002}	{0.000}	{0.051}
Control mean	0.115	0.452	0.115	0.452
Observations	22,604	22,604	22,775	22,775
$R^{2}$	0.532	0.452	0.282	0.280
*P* Near-Peer=Teacher	0.000	0.000	0.000	0.001

This table reports girls’ beliefs immediately after the intervention and 12 months later for those randomized to treatment. Columns (1) and (3) display the percentage of girls reporting a belief that older men are more likely to have HIV and columns (2) and (4) display the percentage of girls reporting a belief that older partners are riskier in general. We have two key belief indicators: knowledge of HIV risks by specific age category as well as more general HIV risk perceptions by broader categories. All specifications are ordinary least squares and include strata fixed effects. SE are in parentheses, 95% CI are in square brackets, and *P*-values are in curved brackets. Results are estimated at the individual level with 95% CI and include robust clustered SE at the school level.

Thus, student beliefs shift when both teachers and near-peers deliver the message and in the same direction, but teachers induce only a partial transfer. This might be due to either the compliance or beliefs margin. It is possible a third of teachers implement and those teachers are just as effective as near-peers at changing beliefs. Alternatively, all teachers might implement but be a third as effective. In the Supplementary material, we explore these dynamics further. Suggestive evidence indicates that fewer teachers implement, but when they do they successfully change beliefs as much as near-peers.

These shifts in beliefs are substantial. Immediately after the intervention, beliefs shift around 4-fold relative to the control mean average; a year after the intervention beliefs change by almost 80%.

We next explore the degree to which beliefs change *as intended*. It is possible that messengers might increase knowledge of older partner risks but not make clear that similar-aged partners are relatively safer, thus partially deviating from the sex education message. For example, teachers might revert to delivering an abstinence message akin to the status quo message: “older partners are riskier, so abstain.” The degree to which the message is implemented as intended is captured through a simple difference capturing relative beliefs on the intensive margin, revealing the *relative risk* of older relative to younger partners. This captures relative risk (eg the risk of older relative to younger men) not just absolute risks (eg only the risk of older men). Figure 1B summarizes results graphically for beliefs about age-specific risk categories. Beliefs shift in a similar direction across arms, with a partial transfer in the teacher arm.

Table 2 summarizes effects on how beliefs shift as intended after 12 months. In this table, we include two key belief indicators: HIV risks by specific age group categories and HIV risk perceptions by more general categories. Effects are also disaggregated for strata with the highest risk-seeking behaviors: junior schools and rural areas. In the near-peer arm, there is an increase in belief transfer about relative risks of around 10% age points (P<0.001) a year later. For the teacher arm, the effect is smaller but significant in several subgroups with up to 5 percentage point (P<0.001) increases in relative risk knowledge transfer. These results suggest that teachers and near-peers can, by and large, both be effective messengers to transfer information.

**Table 2.** Student beliefs shift as intended

	All girls		Junior girls		Rural girls	
	(1)	(2)	(3)	(4)	(5)	(6)
	HIV belief	Risk belief	HIV belief	Risk belief	HIV belief	Risk belief
Near-peer arm	0.099	0.092	0.093	0.085	0.094	0.111
	(0.009)	(0.016)	(0.012)	(0.022)	(0.011)	(0.017)
	[0.080,0.117]	[0.061,0.123]	[0.070,0.116]	[0.042,0.129]	[0.073,0.115]	[0.078,0.144]
	{0.000}	{0.000}	{0.000}	{0.000}	{0.000}	{0.000}
Teacher arm	0.050	0.031	0.018	− 0.004	0.049	0.039
	(0.011)	(0.017)	(0.013)	(0.024)	(0.012)	(0.020)
	[0.029,0.072]	[−0.003,0.065]	[−0.008,0.044]	[−0.053,0.044]	[0.026,0.073]	[−0.001,0.078]
	{0.000}	{0.078}	{0.173}	{0.855}	{0.000}	{0.054}
Control Mean	0.057	0.358	0.061	0.390	0.059	0.342
Observations	21,143	21,143	13,503	13,503	16,130	16,130
*P*: Near-Peer=Teacher	0.000	0.000	0.000	0.000	0.001	0.000

This table reports treatment effects on the percentage of girls who shift their beliefs as intended, such that they learn that older men are more likely to have HIV or be riskier in general than younger men. This captures relative risk (eg the risk of older relative to younger men) not just absolute risks (eg only the risk of older men). This relative risk belief is calculated through the following difference: identification of older partners as risky relative to identification of younger partners as risky. We have two key belief indicators: knowledge of HIV risks by specific age category as well as HIV risk perceptions by broader categories. Results presented are for beliefs 12 months after the intervention. All specifications are ordinary least squares and include strata fixed effects. SE are in parentheses, 95% CI are in square brackets, and *P*-values are in curved brackets. Results are estimated at the individual level with 95% CI and include robust clustered SE at the school level.

### Risky sexual behavior

We next examine effects on risky sexual behavior among adolescent girls. We capture the effect of the sex education message when delivered by near-peers and the relative effect when delivered by teachers.

**Fig. 2.**
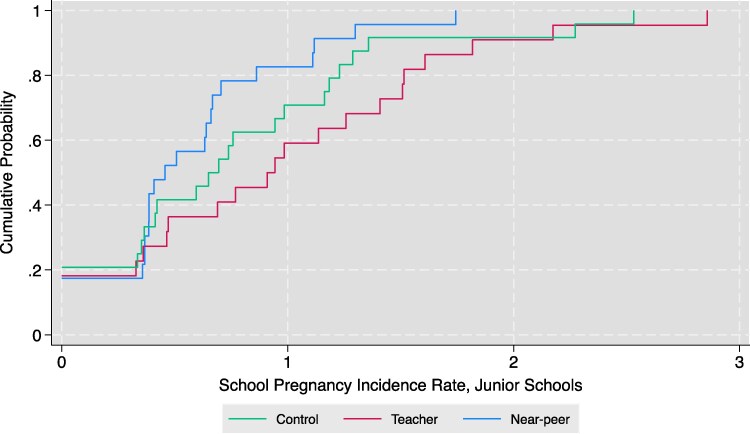
School pregnancy incidence rate by treatment arm. This figure plots the cumulative distribution of treatment effects on school-level pregnancy annual incidence rates for junior school girls by treatment arm (the number of girls pregnant divided by the number of girls in the given grades in the sample and treatment group). School-level pregnancy rates along the *x*-axis are interpreted as the annual pregnancy incidence rate. For example, the number 1 on the *x*-axis refers to 1%.

Figure 2 depicts the cumulative distribution of school-level pregnancy incidence by treatment arm in junior school. Table 3 also shows results for our main pregnancy outcome. Columns (1)–(3) show results from ordinary least squares (OLS) regressions. Pregnancy incidence goes down by 0.206 (P=0.036) percentage points for all girls in the near-peer arm. This represents a 42% reduction in pregnancy incidence relative to the control. Effect sizes are larger for junior schools and in rural areas, with reductions in pregnancy of up to 63%. In contrast, teachers shift the distribution toward higher pregnancy incidence. These effects are statistically significant relative to near-peer results since effects go in opposite directions (although the increase in pregnancy incidence in the teacher arm is not statistically significant when only compared to the control group). We include results of Logistic regressions in columns (4)–(6) which show similar patterns. Figure S5 also shows raw pregnancy incidence rates across treatment arms, again reinforcing the same results and patterns.

**Table 3.** Average treatment effects on pregnancy

	Pregnancy incidence (OLS)			pregnancy incidence (logistic)		
	All girls	Junior girls	Rural girls	All girls	Junior girls	Rural girls
Sex education	−0.206	−0.442	−0.354	−0.498	−0.626	−0.782
	(0.098)	(0.169)	(0.119)	(0.223)	(0.223)	(0.258)
	[−0.399,−0.013]	[−0.781,−0.103]	[−0.588,−0.121]	[−0.936,−0.060]	[−1.063,−0.189]	[−1.286,−0.277]
	{0.036}	{0.012}	{0.003}	{0.026}	{0.005}	{0.002}
Sex education × Teacher	0.273	0.613	0.429	0.659	0.784	0.922
	(0.116)	(0.187)	(0.128)	(0.184)	(0.175)	(0.193)
	[0.045,0.501]	[0.239,0.987]	[0.177,0.681]	[0.298,1.020]	[0.441,1.127]	[0.543,1.301]
	{0.019}	{0.002}	{0.001}	{0.000}	{0.000}	{0.000}
Control mean	0.493	0.737	0.566	0.493	0.737	0.566
Observations	22,903	14,749	17,441	22,903	14,749	17,441

This table reports average treatment effects on annual pregnancy incidence. All specifications include strata dummies and a vector of school-level baseline control variables. We scale the dependent variable by a factor of one hundred for ease of intuitive interpretation of the coefficient as the direct annual incidence rate which is a commonly cited statistic in the literature (eg 0.21 reduction in the pregnancy incidence rate). SE are in parentheses, 95% CI are in square brackets, and *P*-values are in curved brackets. Results are estimated at the individual level with 95% CI and include robust clustered SE at the school level. We include both OLS specifications as well as Logistic specifications given the binary outcome variable. Coefficients in columns 4–6 are interpreted as log odds ratios.

The effects for near-peers are practically significant as well as statistically significant. The effects found represent incidence rates each year rather than prevalence rates which accumulate over time. If one extrapolates the impacts of the program over the course of 5 to 10 years, effects are large in absolute as well as relative terms. Moreover, we conduct a cost-effectiveness comparison in the Discussion section, and find high cost-effectiveness relative to leading alternatives.

These results indicate that, overall, a safer sex education message can be significantly more effective at reducing risky sex among adolescents than status-quo abstinence messaging. This supports the theory that sexual behavior is more likely to change along the intensive margin (type of sex), when students are provided low-risk options such as dating younger partners, rather than abstinence messages, which emphasizes risk avoidance along the extensive margin (sex or no sex). The results in Botswana are consistent in direction and size with results from Kenya 10 years prior (6), indicating that this information mechanism translates across highly diverse contexts. However, the messenger is first-order in determining effect size and direction within Botswana. Students reduce risky sex substantially when they learn from near-peers that same-age partners are safer; however, it seems there is no effect when the message is delivered by teachers, and a plausible backlash with students behaving in a slightly riskier fashion. This result is surprising given that beliefs shift in a similar direction across both near-peer and teacher arms. Instead of producing effects that go in the same direction, risky sex appears to diverge in opposite directions across messengers. In the Figures S6–S8 and Tables S3–S8, we explore suggestive messenger mechanisms to help explain these results.

## Discussion

Governments worldwide provide sex education in schools. A growing body of evidence exists on messages that are most effective, such as providing safe sex options rather than promoting abstinence. However, less is known about the messenger. In this article, we test the importance of both the message and the messenger.

We find that when a sex education message is delivered by near-peers, it can reduce pregnancy by 40 to 63% in Botswana. However, results show that the messenger can make or break the sex education message. When near-peers deliver the message, pregnancy decreases while when teachers deliver the message there is a null effect and a plausible increase in pregnancy. Thus, we find that both the message and, crucially, the messenger matter.

We explore various messenger mechanisms to explain our results in the Supplementary material. Teachers appear less likely to comply and deliver the message than near-peers, but conditional on compliance, teachers seem to be as effective as near-peers at changing beliefs. This indicates that teachers can be effective messengers for their core business: transferring information. This also suggests that results on risky sex are driven by factors beyond beliefs, since beliefs change similarly and converge, while risky sexual behaviors diverge. An analysis of mechanisms suggests that the messenger determines whether students listen to the message and respond in kind or results in the information being ignored or possibly backfiring in order to demonstrate a sense of agency. The Supplementary material includes a conceptual framework and set of results exploring this possibility.

We calculate cost-effectiveness for the near-peer delivery of sex education where we find positive effects. The sex education message and mode of delivery are cheap since the intervention is one-hour and near-peers are paid a volunteer stipend, similar to most government national service programs. The marginal cost of reaching the average child is $1.08. We calculate cost-effectiveness for girls in junior school: $76.92 per pregnancy averted, assuming pregnancies averted persist at least a few years. The high cost-effectiveness for girls in junior school is driven by large reductions in pregnancy at junior school and low marginal costs to reach a child. These effects can be extrapolated to reductions for HIV infections averted under certain assumptions. Of note, the relationship between how many HIV infections occur per coital act relative to pregnancies is unresolved, so these estimates are merely suggestive.^d^ We use a conservative estimate based on the available literature where 100 pregnancies averted corresponds to 5 HIV infections averted. Our estimates suggest that for girls in junior school it costs $1,538 per instance of HIV infection averted.

As a comparison, a recent review of other highly effective HIV prevention methods found the median HIV infection averted cost $1,144 for prevention of mother-to-child transmission, $2,965 for voluntary male circumcision, $3,576 for conditional cash transfers, $7,903 for treatment-as-prevention interventions, and $13,267 for pre-exposure prophylaxis (32). This comparison reveals that sex education when delivered by near-peer messengers can be similarly cost-effective to alternative leading approaches to prevent risky sex and, in some cases, up to an order of magnitude more cost-effective.

In addition to contributing to the sex education literature highlighted in the Introduction section, our results contribute to a few additional literatures. We contribute to a literature on the role of information in changing beliefs and behaviors. Information has been shown to be consequential in multiple contexts including education (33), sexual behavior (6), HIV testing (12), gender attitudes (9), demand for contraceptives (8), fertility (7), and intimate partner violence (10). We contribute to this literature reinforcing that information can cost-effectively change risky behavior, beyond multiple other factors which influence risky sex, such as the legal environment (34) as well as financial constraints and incentives (35, 36). Moreover, we find information can change behavior in multiple directions, with the messenger influencing the direction.

We also relate to the literature on persuasion. A theoretical literature exists on both the type of information and the sender of information in the persuasion process (37, 38). In our study we refer to the sender of information as the messenger. The results in this trial suggest a persuasion rate of 95% when information is delivered by near-peers.^e^ This persuasion rate ranks in the top percentile in a review by DellaVigna and Gentzkow (18) and builds on recent studies exploring the persuasiveness of various messengers in the context of immigration (20) and public health messages (17, 19). The results in this study provide experimental evidence that the messenger is a central component of effective persuasion.

Finally, a growing literature examines external validity across contexts. This literature includes frameworks (39), extrapolation across contexts (40), and study aggregation (41). Research testing scalability across settings can substantially enhance scientific reliability (42). In our case, the effectiveness of a similar sex education message across both Botswana and Kenya, with up to 40 and 28% reductions in pregnancy, respectively, is striking since the trials took place across highly diverse contexts. The Kenya trial took place from 2003 to 2005 in a low-income context and when HIV medication availability was limited. The Botswana trial took place from 2014 to 2015 in an upper-middle income setting with widely available HIV medication. It is not clear ex ante if the effects of the sex education message will replicate. For example, the cost of HIV risks posed by older partners might have been higher in Botswana since average HIV rates were five times higher than in the Kenya study. On the other hand, the cost of HIV might have been lower since in 2014–2015 in Botswana there was free and widely available antiretroviral medication and HIV was no longer lethal.

Our results suggest that, despite significant scope for heterogeneity across contexts, the effectiveness of the sex education message can generalize across countries from Kenya to Botswana and across time 10 years later. These results reinforce that information does matter and can reduce risky sex, although numerous other factors might drive risky sex. In addition, the results of this study show that the direction of effects turn less on geography and associated covariates, which often receive significant attention in external validity debates, and more on implementation modalities, such as the messenger of a message, within the exact same geography.

Our findings have significant policy implications. Teachers are often the default messenger for sex education. Teachers are respected figures in the community and a fixture of the education system. However, complementary government schemes exist which could be leveraged to deliver sex education. Over 15 countries in sub-Saharan Africa have national service programs (3). Similar schemes exist throughout the world. For example, AmeriCorps in the United States places young volunteers in over 12,000 schools throughout the country.

Our results suggest that while the default messenger for sex education, teachers, can be effective for transferring information and might be best placed to provide basic sex education facts, such as what HIV is, they might not persuade students to reduce risky sex. In contrast, near-peers can reduce pregnancy by over 40%. These results reveal the potential for governments to deliver large welfare gains by deploying national service schemes to deliver sex education at scale. More generally, the results in this trial show that messengers can be first-order in determining whether a given message has the desired effect. Policymakers can leverage this insight to optimize messengers of messaging campaigns and cost-effectively influence behavior across multiple domains.

Our study has several limitations. We do not examine all types of messengers. Given the importance of the messenger in our study, future research might provide causal evidence on the effectiveness of other possible messengers, such as parents. Future research could also compare alternative mediums (as well as messengers), such as online classes, TV, or radio campaigns (7, 43, 44). In addition, while we include a careful and comprehensive follow-up over a year later, future research could conduct even longer-run follow-up and analysis.

## Materials and methods

### Baseline data

Baseline data were collected using surveys as well as administrative records obtained from the Ministry of Education and the Botswana Education Council. Baseline surveying in schools took place from August to November 2014 in all 343 schools. A short, anonymous one-page survey was conducted where students were asked to answer 20 questions covering demographic information, knowledge, attitudes and self-reported sexual behavior. All surveys were completed prior to the intervention.

Administrative data were obtained from the Ministry of Education on enrollment, dropouts, and dropout rationale (including pregnancy, truancy, performances, illness and others) in 2012 for all 343 schools in our final sample. These data were collected using written forms sent to all school heads which are in turn completed and returned via post to the Ministry of Education. These data are compiled at the school level. Administrative data were obtained from the Botswana Education Council on test score performance from the 2014 Primary School Leaving Examination (PSLE) and the Junior Certificate Examination (JCE), both standardized national examinations conducted annually. These data contain absolute test score performance, percent of students achieving grade A, B, or C and demographic information on age and gender composition at the school level.

The baseline data serve multiple purposes: checks for balance to verify the integrity of the randomization as well as descriptive information on the profiles of messengers and schools where the intervention was delivered.

### Process monitoring data

In addition to the baseline survey, surveys were conducted immediately after the intervention to capture immediate shifts in beliefs in both treatment arms. All near-peer arm schools participated in this survey. Around a third of schools in the teacher arm submitted student responses with this information. The Supplementary material explores these data in more detail, showing submission rates were largely unbiased and representative across treatment arms. Moreover, implementation fidelity—specifically procedural fidelity—among teachers is captured using three core measures: attendance at training, commitment card signing, and returning of students’ responses postintervention. All measures are imperfect but plausibly capture various bounds on implementation in line with the expected intervention delivery procedure.

### Endline data

An in-school survey similar to the baseline was conducted in all classes and schools. The endline school data collection commenced in mid-August 2015 and was completed by the end of December 2015.

Surveying took place within the same schools surveyed at baseline at the next grade level assuming standard progression. Grade 6 students progressed to grade 7 in primary schools, and grade 8 and 9 students in junior school progressed to grades 9 and 10, respectively. We have an extremely high almost universal follow-up rate—one of the features of the paper—successfully surveying nearly all students (roughly 42,000 students and about 22,000 girls) assessed at baseline and following up 99% of all girls identified to be pregnant a year later. Given this high response rate, concerns over nonresponse bias are minimal, and we do not impute any missing data.

To avoid any potential surveyor effects at endline, the same set of surveyors conducted endline surveys across all treatment arms and surveyors were assigned randomly to each school to ensure survey responses were unbiased across treatment arms. The average difference between the survey date and the start of endline ranged from 13 to 13.5 days across all arms and was statistically equivalent. Adherence to instructions and the percent of responses missing were also statistically equivalent across all three arms. The survey was a short, anonymous, two-page survey where students were asked to answer 34 questions covering student demographics, knowledge, attitudes and self-reported sexual behavior. Surveys were split into “female” surveys and “male” surveys to tailor gender-specific questions. For example, question 27 in the male survey reads “Have you ever made anyone pregnant?” whereas in the female version it reads “Have you ever been pregnant?”

In terms of beliefs, we focus our analysis on two key belief indicators: knowledge of specific HIV risks by disaggregated age category as well as HIV risk perceptions for broader age categories. The first question asks “the age group of men most likely to have HIV is” and includes age categories “10–19,” “20–29”, “30–39,” “40–49”, and “50 and above” as answer choices. The second question asks “who do you think has the highest risk of infecting you with the HIV/AIDS virus” and includes answer choices “your age mates” “other young people” “older people” or “I don’t know.” These beliefs are calculated and reported on their own, and we also construct an index which takes a simple difference of knowing the risk of older men or older age categories relative to knowing the risk of younger men or younger age categories, to ensure both ends of the spectrum shift. This captures changes in relative risk knowledge, in addition to changes in absolute risk knowledge.

In addition, a “roll call” method was used to collect data on girls missing from school using baseline registers of students present at baseline. If a girl was missing, classmates were asked to identify the reason, including whether she had fallen pregnant. In rural communities, it is common knowledge if a girl has dropped out due to pregnancy, a visible and biological outcome. Legally, all girls who fall pregnant are required to withdraw from school for a full year suggesting that this measure captures nearly all pregnancies. This provision is detailed in Section 34 of the Botswana Education Act.

We confirmed the roll call data by also asking school administrators to identify any girls missing from school due to pregnancy. School administrator surveys were triangulated real-time with the roll call data. The lead enumerator at each school was instructed to check if both surveys aligned. A maximum of the roll call and administrator data were taken to identify pregnancies. The survey also asked demographic information on the school administrator’s age, credentials, and years teaching, as well as overall enrolment numbers.

Our measure of pregnancy is computed using a maximum of roll-call and school administrator data at the school. This school-based data are our primary outcome. In the Supplementary material, we describe the verification of our main measure of pregnancy using these data. Over 90% of pregnancies identified at the school were confirmed during at-home visits. This outcome provides an objective measure of behavior and a direct comparison to studies which uses similar measurements to identify pregnancy (6, 14). At-home visits validate and increase confidence in use of the school-based data, as well as provide additional texture on the results.

We use pregnancy as a measure of risky sex. It is important to note that pregnancy rates do not correspond directly to HIV rates. For example, while unprotected sex is a root cause of both HIV and pregnancy, one can have unprotected sex and still not contract HIV if viral loads are low due to medication usage. While we considered collecting data on HIV directly, we could not collect HIV data due to high costs and low feasibility of conducting repeat HIV testing. Moreover, there are ethical concerns when conducting sensitive HIV testing, which would also require ensuring sufficient infrastructure to provide follow-up counseling once students learn their results. Thus, we use pregnancy as a measure of risky sex since it is feasible to collect at scale, it has been used in other prominent studies in the literature, and it is a consequential outcome in its own right, but results cannot be assumed to translate entirely to HIV rates.

Study protocols and IRB were approved by the Ministry of Education (reference: DEPRS 7/1/5/ XII (23), and the Ministry of Health HRDC (protocol number: 00756) in Botswana and MIT (protocol number: 1408006559). This trial is registered in the AEA trial registry at AEARCTR-0007624. Consent was obtained by all participants prior to conducting the surveys and it was made explicit that participants could opt out of answering specific questions.

### Empirical strategy and statistical analysis

#### Identifying effects on student beliefs

Random assignment enables identification of causal effects of the sex education message when delivered by near-peers and teachers. We run an ordinary least squares regression using the following specification:


(1) $${\mathrm{belief}}_{ij}=\alpha +\beta_{1}\;{\mathrm{peer}}_{j}+\beta_{2}\;{\mathrm{teacher}}_{j}+\delta_{s}+\epsilon_{ij},$$

where ${\mathrm{beliefs}}_{ij}$ refers to a belief outcome—such as HIV knowledge—for individual *i* in school *j*. The terms *peer* and *teacher* denote a dummy variable which takes on a value of 1 when a school is assigned to each arm and 0 otherwise. $\delta_{s}$ is a dummy for strata fixed effects including region and school level (primary or junior). SE are clustered at the school level, the unit of randomization. Our primary analysis focuses on effects for girls to relate beliefs to pregnancy and sexual behavior outcomes.

### Intention-to-treat estimates for risky sexual behavior

We capture the effect of the sex education message when delivered by near-peers and the relative effect on risky sexual behavior when delivered by teachers. We estimate intent-to-treat effects using both linear and logistic regression as follows:


(2) $$Y_{ij}=\alpha +\beta_{1}\;{\mathrm{SexEd}}_{j}+\beta_{2}\;\mathrm{SexEd}\;*\;{\mathrm{teacher}}_{j}+\gamma X_{j}+\delta_{s}+\epsilon_{ij},$$

where $Y_{ij}$ is a sexual behavior outcome for individual *i* in school *j*. ${\mathrm{SexEd}}_{j}$ refers to the sex education message and takes on the value 1 in both treatment arms and 0 otherwise. $X_{s}$ denotes a vector of school-level baseline control variables to enhance statistical power and precision. These variables include: male and female enrollment, gender composition, average age, baseline dropouts, self-reported rates of pregnancy, the percent of girls in the class reported to be pregnant, the percent of girls in the class reported to have older boyfriends, and a baseline literacy index. Results are also disaggregated for junior schools and rural areas.

### Statistical power analysis

We include a power calculation to inform the degree to which our study design was powered to detect effects. We assume the following: power=80%, α=0.05; ρ=0.1; cluster size=100 schools per arm; students per cluster=100; baseline school-level mean pregnancy incidence=0.3; and SD of school-level mean pregnancy incidence=0.65. We base school-level pregnancy on data collected from the Ministry of Education at baseline. Under these assumptions, we are powered to detect a minimum detectable effect (MDE) of 0.09, picking up reductions in pregnancy incidence from an estimated baseline mean of 0.3 to 0.21. In our results, we observe larger effects sizes than the MDE in our power calculations and can detect these effects as expected.

## Supplementary Material

pgag183_Supplementary_Data

## Data Availability

Deidentified data used for analysis in the article will be made available on a Github repository here: https://github.com/angristnoam/messengermatters.

## Notes

a The role of messengers who are more similar to the receiver of their message, such as peers or near-peers, has been shown to be important for agricultural technology adoption (45) and demand for vaccines (17).

b Near-peer is a term common in public health (46). While peers are often the same age as recipients of information and in the same social network (47), near-peers are typically similar in age yet slightly older, are often out of the direct social network of the recipient of information, and are aspirational role models.

c A high-profile case of sex education messaging includes the President’s Emergency Plan for AIDS Relief (PEPFAR) which has spent $80 billion (USD) on the HIV response. PEPFAR provides life-saving HIV treatment for millions; however, the evidence on PEPFAR-related sex education is mixed. Lo et al. (48) find that the dominant sex education message promoted by PEPFAR in its first decade, abstinence, had no effect on reducing risky sex. Numerous experimental studies in the public health literature have found similar results (49, 50). The growing evidence on effective sex education messages has shifted policy: over 80% of governments have now adopted information shown to reduce high-risk sexual behavior in national curricula, such as comprehensive safe sex strategies over abstinence-only messages (15). Consortia have formed to prioritize these types of messages, such as the “Ministerial Commitment for Adolescent and Sexual Reproductive Health” among 20 countries in Africa to deliver comprehensive sexuality education. The same level of effort to rigorously study effective messengers could be similarly influential.

d This is in part because mapping the relationship to the root source of both, instances of unprotected sex, is highly context dependent. For example, the risk of HIV infection conditional on an instance of unprotected sex can vary 10-fold depending on the stage of HIV infection (51, 52). Other factors can also drive variation in infectiousness such as whether relationships are monogamous and whether male partners are circumcised.

e We calculate this in line with DellaVigna and Gentzkow (18): f=100*  $\left(y_{T}-y_{C})/(e_{T}-e_{C})*1/(1-y_{0}\right)$ for a binary outcome, treatment group *T*, and control group *C*. $e_{i}$ is the share of group i receiving the message, $y_{i}$ is the share of group i adopting the behavior of interest, and $y_{0}$ is the share that would adopt the behavior if there were no message (approximated by $y_{c}$ where $y_{0}$ is not observed). The most conservative estimate of $y_{T}-y_{C}$ for near-peers is estimated by a reduction in pregnancy incidence of 0.206% (Table 3) $e_{T}-e_{C}$ can be estimated by the percentage of students who learn older partners have the highest rate of HIV of 44.2% (Table 1), and $1-y_{0}$ can be estimated as the share of the population that falls pregnant in the control group in a given year of roughly half a percent (control mean in Table 3). This produces a persuasion rate of 95.

## References

[1] van Schalkwyk C, Mahy M, Johnson LF, Imai-Eaton JW. 2024. Updated data and methods for the 2023 UNAIDS HIV estimates. J Acquir Immune Defic Syndr. 95:e1–e4. 10.1097/QAI.0000000000003344 PMC10769173 38180734

[2] UNAIDS . 2018. Global HIV & AIDS statistics–2018 fact sheet. Geneva, Switzerland. Technical Report.

[3] Bodley-Bond C, Cronin K. National youth service, employability, entrepreneurship and sustainable livelihoods: overview of the national youth service landscape in Sub-Saharan Africa. Innovation in Civic Participation, 2013.

[4] Oster E . 2012. HIV and sexual behavior change: why not Africa? J Health Econ. 31:35–49. 22277285 10.1016/j.jhealeco.2011.12.006

[5] Stephenson JM, et al 2004. Pupil-led sex education in England (ripple study): cluster-randomised intervention trial. Lancet. 364:338–346. 15276393 10.1016/S0140-6736(04)16722-6

[6] Dupas P . 2011. Do teenagers respond to HIV risk information? Evidence from a field experiment in Kenya. Am Econ J Appl Econ. 3:1–34.22199993

[7] Ferrara EL, Chong A, Duryea S. 2012. Soap operas and fertility: evidence from Brazil. Am Econ J Appl Econ. 4:1–31.

[8] Miller G, Paula AD, Valente C. 2020. Subjective expectations and demand for contraception. National Bureau of Economic Research. Technical Report w27271.

[9] Dhar D, Jain T, Jayachandran S. 2022. Reshaping adolescents’ gender attitudes: evidence from a school-based experiment in India. Am Econ Rev. 112:899–927.

[10] Shah M, Seager J, Montalvao J, Goldstein M. 2023. Sex, power, and adolescence: intimate partner violence and sexual behaviors. National Bureau of Economic Research. Technical Report w31624.

[11] Kirby DB . 2008. The impact of abstinence and comprehensive sex and STD/HIV education programs on adolescent sexual behavior. Sex Res Soc Policy. 5:18–27.

[12] Paula AD, Shapira G, Todd PE. 2014. How beliefs about HIV status affect risky behaviors: evidence from Malawi. J Appl Econom. 29:944–964.

[13] Walque DD . Risking your health: causes, consequences, and interventions to prevent risky behaviors. World Bank, 2014.

[14] Duflo E, Dupas P, Kremer M. 2015. Education, HIV, and early fertility: experimental evidence from Kenya. Am Econ Rev. 105:2757–2797. 26523067 10.1257/aer.20121607 PMC4624413

[15] UNESCO . 2015. Emerging evidence, lessons and practice in comprehensive sexuality education: a global review. Paris, France. Technical report.

[16] Dodd S, et al 2022. School-based peer education interventions to improve health: a global systematic review of effectiveness. BMC Public Health. 22:2247. 36461024 10.1186/s12889-022-14688-3 PMC9719233

[17] Alsan M, Eichmeyer S. 2021. Experimental evidence on the effectiveness of non-experts for improving vaccine demand. NBER Working Paper. Technical Report 28593. 10.1257/pol.20210393 PMC10907065 38433953

[18] DellaVigna S, Gentzkow M. 2010. Persuasion: empirical evidence. Annu Rev Econom. 2:643–669.

[19] Torres C, et al 2021. Effect of physician-delivered COVID-19 public health messages and messages acknowledging racial inequity on black and white adults’ knowledge, beliefs, and practices related to COVID-19: a randomized clinical trial. JAMA Netw Open. 4:e2117115. 34259846 10.1001/jamanetworkopen.2021.17115 PMC8280971

[20] Afrouzi H, Arteaga C, Weisburst EK. 2024. Is it the message or the messenger? Examining movement in immigration beliefs. J Polit Econ Microecon. 2:244–297.

[21] Godlonton S, Munthali A, Thornton R. 2016. Responding to risk: circumcision, information, and HIV prevention. Rev Econ Stat. 98:333–349.

[22] Kerwin J . 2020. Scared straight or scared to death? Fatalism in response to disease risks. Technical Report.

[23] List JA . 2024. Optimally generate policy-based evidence before scaling. Nature. 626:491–499. 38356064 10.1038/s41586-023-06972-y

[24] Botswana S . 2013. Botswana aids impact survey IV (BAIS IV): 2013 summary results. Gaborone, Botswana. Technical Report.

[25] Luke N, Kurz K. 2002. Cross-generational and transactional sexual relations in sub-Saharan Africa. International Center for Research on Women. Technical report.

[26] Adams A, Andrew A. 2019. Preferences and beliefs in the marriage market for young brides. IFS Working Papers. Technical Report W19/05.

[27] Makhema J, et al 2019. Universal testing, expanded treatment, and incidence of HIV infection in Botswana. N Engl J Med. 381:230–242. 31314967 10.1056/NEJMoa1812281 PMC6800102

[28] Ussery F, et al 2022. HIV incidence in Botswana rural communities with high antiretroviral treatment coverage: results from the Botswana Combination Prevention Project, 2013–2017. J Acquir Immune Defic Syndr. 91:9–16. 35537094 10.1097/QAI.0000000000003017 PMC9388617

[29] Ministry of Basic Education . 2016. Second Botswana youth risk behavioural and biological surveillance report. Gaborone, Botswana. Technical Report.

[30] UNESCO Institute for Statistics (UIS) . 2024. Data for Botswana—education statistics, 2014. http://data.uis.unesco.org. Accessed: October 17.

[31] Statistics Botswana . 2012. Education Statistics Report 2012.

[32] Sarkar S, et al 2019. Cost-effectiveness of HIV prevention interventions in sub-Saharan Africa: a systematic review. EClinicalMedicine. 10:10–31. 31193863 10.1016/j.eclinm.2019.04.006 PMC6543190

[33] Jensen R . 2010. The (perceived) returns to education and the demand for schooling. Q J Econ. 125:515–548.

[34] Cunningham S, Shah M. 2018. Decriminalizing indoor prostitution: implications for sexual violence and public health. Rev Econ Stud. 85:1683–1715.

[35] Gertler P, Shah M, Bertozzi SM. 2005. Risky business: the market for unprotected commercial sex. J Polit Econ. 113:518–550.

[36] Bjorkman Nyqvist M, Corno L, Walque DD, Svensson J. 2018. Incentivizing safer sexual behavior: evidence from a lottery experiment on HIV prevention. Am Econ J Appl Econ. 10:287–314.

[37] Mullainathan S, Schwartzstein J, Shleifer A. 2008. Coarse thinking and persuasion. Q J Econ. 123:577–619.

[38] Kamenica E, Gentzkow M. 2011. Bayesian persuasion. Am Econ Rev. 101:2590–2615.

[39] Duflo E, Glennerster R, Kremer M. 2007. Using randomization in development economics research: a toolkit. In Handbook of development economics. p. 3895–3962. https://www.sciencedirect.com/science/chapter/handbook/pii/S1573447107040612?casa_token=TsKj8hA7AC4AAAAA:5vGzgujy8gV8g4L0Y4djKwfb5yzvrUWlhewZgrtmR643KKQSLUaHX6izvNT-5_FnXWZo3s7ndF0

[40] Allcott H . 2015. Site selection bias in program evaluation. Q J Econ. 130:1117–1165.

[41] Meager R . 2022. Aggregating distributional treatment effects: a Bayesian hierarchical analysis of the microcredit literature. Am Econ Rev. 112:1818–1847.

[42] Maniadis Z, Tufano F, List JA. 2017. To replicate or not to replicate? Exploring reproducibility in economics through the lens of a model and a pilot study. Econ J. 127:F209–F235.

[43] Chong A, Gonzalez-Navarro M, Karlan D, Valdivia M. 2020. Do information technologies improve teenagers’ sexual education? Evidence from a randomized evaluation in Colombia. World Bank Econ Rev. 34:371–392.

[44] Glennerster R, Murray J, Pouliquen V. 2021. The media or the message? Experimental evidence on mass media and modern contraception uptake in Burkina Faso. Center for the Study of African Economies Working Paper. Technical report.

[45] BenYishay A, Mushfiq Mobarak A. 2019. Social learning and incentives for experimentation and communication. Rev Econ Stud. 86:976–1009.

[46] Saul J, et al 2018. The dreams core package of interventions: a comprehensive approach to preventing HIV among adolescent girls and young women. PLoS One. 13:e0208167. 30532210 10.1371/journal.pone.0208167 PMC6285267

[47] Sacerdote B . 2001. Peer effects with random assignment: results for Dartmouth roommates. Q J Econ. 116:681–704.

[48] Lo NC, Lowe A, Bendavid E. 2016. Abstinence funding was not associated with reductions in HIV risk behavior in sub-Saharan Africa. Health Affairs. 35:856–863. 27140992 10.1377/hlthaff.2015.0828

[49] Gallant M, Maticka-Tyndale E. 2004. School-based HIV prevention programmes for African youth. Soc Sci Med. 58:1337–1351. 14759680 10.1016/S0277-9536(03)00331-9

[50] Paul-Ebhohimhen VA, Poobalan A, van Teijlingen ER. 2008. A systematic review of school-based sexual health interventions to prevent STI/HIV in sub-Saharan Africa. BMC Public Health. 8:1–4. 18179703 10.1186/1471-2458-8-4 PMC2248569

[51] Hayes RJ, White RG. 2006. Amplified HIV transmission during early-stage infection. J Infect Dis. 193:604–605. 16425144 10.1086/499606

[52] Déirdre Hollingsworth T, Anderson RM, Fraser C. 2008. HIV-1 transmission, by stage of infection. J Infect Dis. 198:687–693. 18662132 10.1086/590501

