# Supplementary material for: The messenger matters: Behavioral responses to sex education in a cluster randomized trial
Source: PNAS Nexus. 2026 May 22;5(6):pgag183. doi: 10.1093/pnasnexus/pgag183 (PMC13234950; doi:10.1093/pnasnexus/pgag183)
Supplement: pgag183_Supplementary_Data [file pgag183_supplementary_data.pdf]

The Messenger Matters: Behavioral Responses to Sex Education in a Cluster Randomized Trial

Noam Angrist, Gabriel Anabwani

Appendix A: Supplementary Figures and Tables for the Main Text

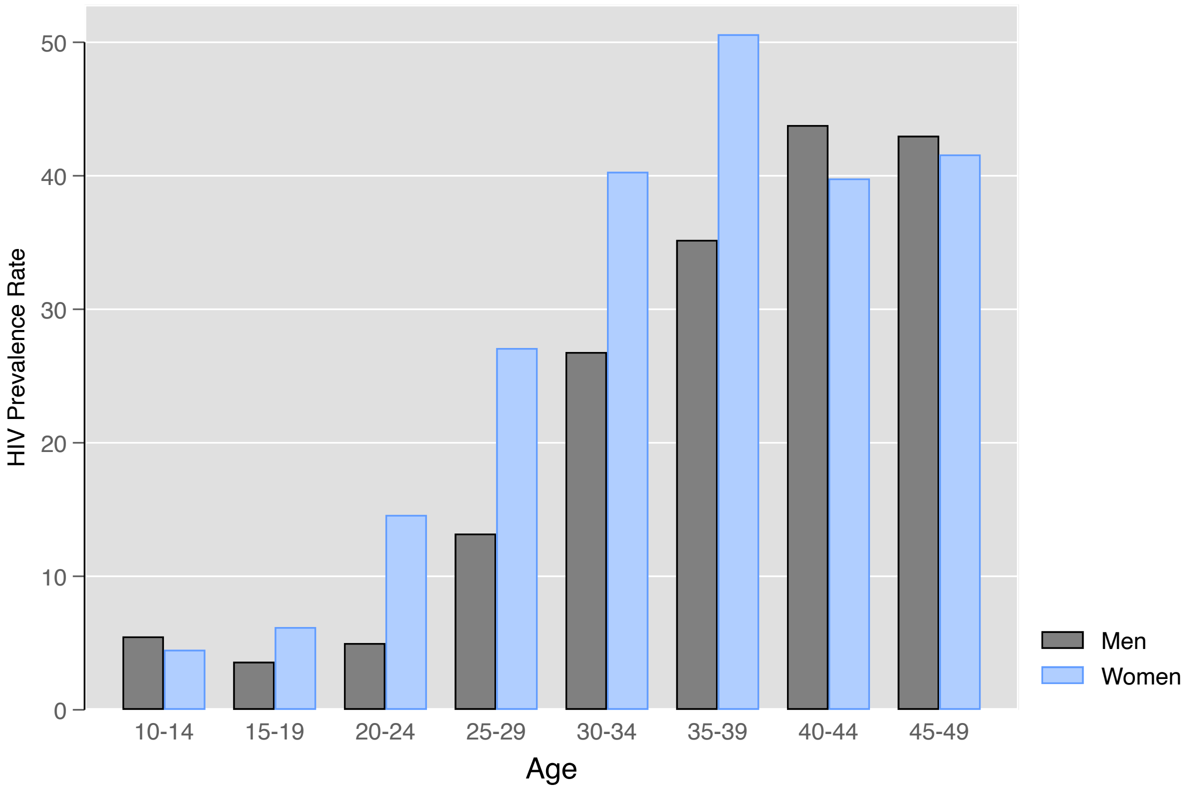

Fig. S1. HIV prevalence by gender and age in Botswana *Source:* Botswana AIDS Impact Survey (2013). The BAIS survey is a nationally representative survey conducted in Botswana every few years. HIV prevalence rates are the percent of all persons in the given age group who have HIV.

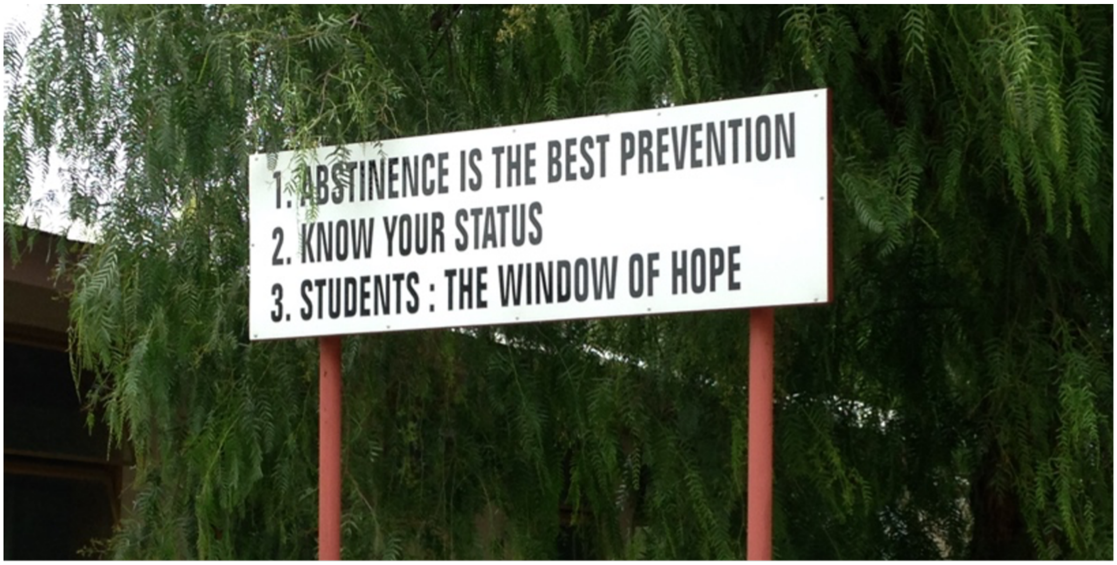

Fig. S2. Typical Sign at a School

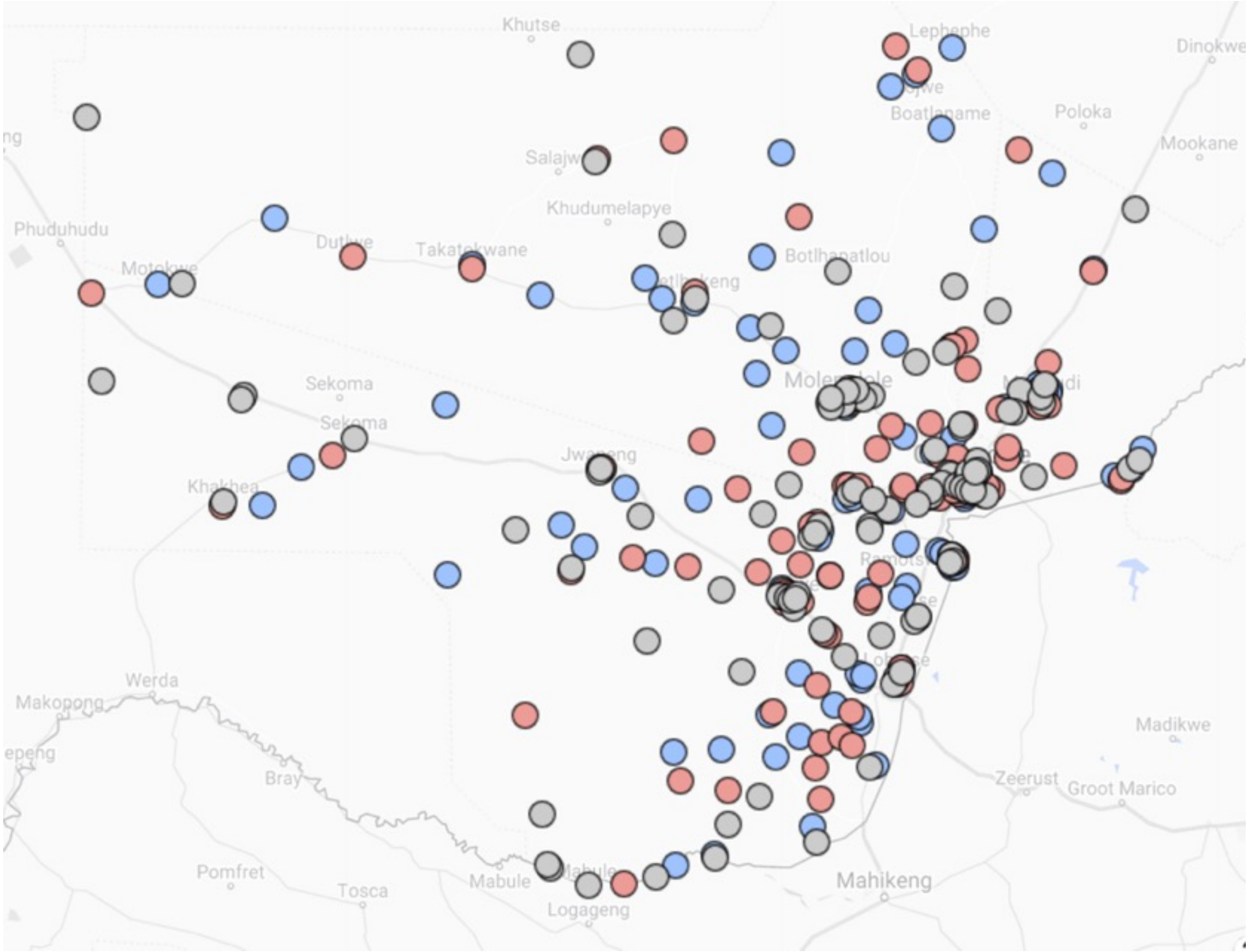

Fig. S3. Gray = Control, Red = Teacher, Blue = Near-Peer.

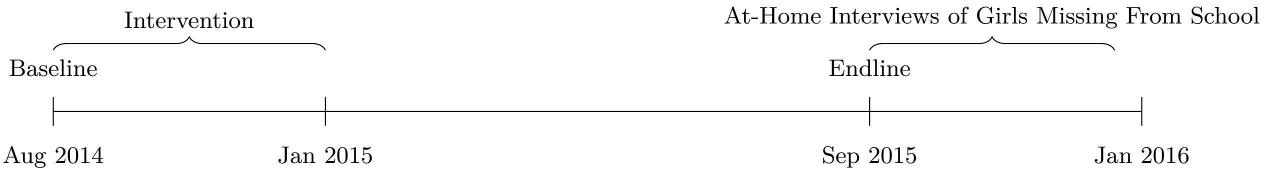

Fig. S4. Timeline

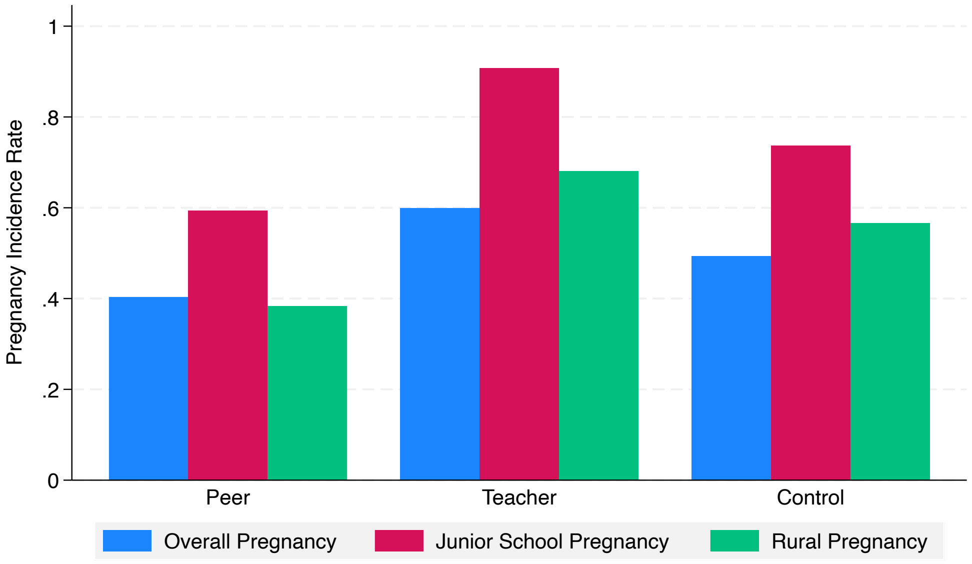

Fig. S5. Raw Pregnancy Incidence Rates. This figure shows raw pregnancy rates based on simple means. Effects are similar to treatment effects estimated from the the full empirical specification including strata fixed effects and controls.

Table S1. Messenger Profiles

	Mean	SD	Min	Max
Panel A: Teachers				
Female	0.79	0.41	0.00	1.00
Age (Years)	43.29	5.70	25.00	50.00
Years of Teaching Experience	20.87	6.98	1.75	36.75
Highest Degree Attained - Bachelors	0.13	0.34	0.00	1.00
Highest Degree Attained - Diploma	0.60	0.49	0.00	1.00
Panel B: Near-Peers				
Female	0.88	0.34	0.00	1.00
Age (Years)	24.56	2.68	19.00	30.00
Years of Teaching Experience	0.00	0.00	0.00	0.00
Highest Degree Attained - Bachelors	0.33	0.49	0.00	1.00
Highest Degree Attained - Diploma	0.27	0.46	0.00	1.00

Notes: This table reports descriptive statistics by messenger type. We include standard deviations, minimum and maximum descriptive statistics for both continuous variables as well as for dummy variables to provide a parallel structure, although in the latter case standard deviations add limited information.

Table S2. Baseline Summary Statistics and Balance

Variable	(1) Peer N	(1) Peer Mean/(SE)	(2) Teacher N	(2) Teacher Mean/(SE)	(3) Control N	(3) Control Mean/(SE)	F-test for balance across all groups N	F-stat/P-value	Pairwise t-test (1)-(2) N	(1)-(2) Mean difference	(1)-(3) N	(1)-(3) Mean difference	(2)-(3) N	(2)-(3) Mean difference
Female	113	0.502<br>(0.008)	114	0.492<br>(0.007)	116	0.491<br>(0.008)	343	0.614<br>0.542	227	0.010	229	0.011	230	0.001
Age (Years)	113	12.783<br>(0.099)	114	12.756<br>(0.098)	116	12.725<br>(0.099)	343	0.089<br>0.915	227	0.027	229	0.059	230	0.032
Class Size	113	29.125<br>(0.768)	114	29.310<br>(0.817)	116	30.093<br>(0.806)	343	0.417<br>0.659	227	-0.185	229	-0.968	230	-0.783
School Size	113	124.301<br>(14.313)	114	131.860<br>(14.275)	116	133.259<br>(13.921)	343	0.115<br>0.891	227	-7.559	229	-8.958	230	-1.399
School Absenteeism	113	0.042<br>(0.005)	114	0.035<br>(0.004)	116	0.041<br>(0.005)	343	0.592<br>0.554	227	0.007	229	0.001	230	-0.006
Grade 6	113	0.760<br>(0.037)	114	0.767<br>(0.036)	116	0.760<br>(0.036)	343	0.012<br>0.988	227	-0.007	229	0.000	230	0.007
Grade 8	113	0.101<br>(0.019)	114	0.101<br>(0.019)	116	0.106<br>(0.019)	343	0.021<br>0.979	227	0.000	229	-0.005	230	-0.005
Grade 9	113	0.100<br>(0.019)	114	0.098<br>(0.018)	116	0.098<br>(0.018)	343	0.004<br>0.996	227	0.002	229	0.002	230	-0.000
Highest Male HIV Risk Group	113	0.106<br>(0.006)	114	0.113<br>(0.007)	116	0.107<br>(0.005)	343	0.373<br>0.689	227	-0.007	229	-0.001	230	0.006
Older Partners Are Riskier	113	0.420<br>(0.010)	114	0.419<br>(0.010)	116	0.423<br>(0.010)	343	0.053<br>0.948	227	0.001	229	-0.004	230	-0.004
Ever Had Sex	113	0.136<br>(0.012)	114	0.129<br>(0.012)	116	0.112<br>(0.009)	343	1.359<br>0.258	227	0.007	229	0.024	230	0.018
Ever Had Sex - Older	113	0.071<br>(0.007)	114	0.065<br>(0.007)	116	0.057<br>(0.006)	343	1.034<br>0.357	227	0.006	229	0.013	230	0.007
Ever been Pregnant	113	0.078<br>(0.007)	114	0.062<br>(0.006)	116	0.061<br>(0.007)	343	2.002<br>0.137	227	0.015	229	0.016	230	0.001
Girls Ever Pregnant in Class	113	0.134<br>(0.063)	114	0.036<br>(0.010)	116	0.094<br>(0.038)	343	1.322<br>0.268	227	0.098	229	0.040	230	-0.058
Grade (BCE)	112	51.964<br>(0.729)	113	52.777<br>(0.749)	114	52.796<br>(0.772)	339	0.399<br>0.671	225	-0.813	226	-0.833	227	-0.019
% A (BCE)	112	0.070<br>(0.007)	113	0.080<br>(0.007)	114	0.079<br>(0.009)	339	0.478<br>0.621	225	-0.010	226	-0.009	227	0.001
% B (BCE)	112	0.134<br>(0.009)	113	0.144<br>(0.008)	114	0.150<br>(0.008)	339	0.880<br>0.416	225	-0.010	226	-0.015	227	-0.006
% C (BCE)	112	0.362<br>(0.011)	113	0.364<br>(0.011)	114	0.364<br>(0.011)	339	0.006<br>0.994	225	-0.001	226	-0.002	227	-0.000
Total Enrollment (MOE)	113	452.248<br>(22.090)	114	479.342<br>(24.525)	116	489.638<br>(24.163)	343	0.666<br>0.514	227	-27.094	229	-37.390	230	-10.296
Preg Dropout % (MOE)	113	0.154<br>(0.041)	114	0.108<br>(0.035)	116	0.287<br>(0.166)	343	0.842<br>0.432	227	0.046	229	-0.133	230	-0.179
F-test of joint significance (P-value)										0.807		0.806		0.837
F-test, number of observations										225		226		227

Notes: This table reports summary statistics and p-values on a joint F-test across baseline characteristics from survey and administrative data for all students at the school level. Administrative data on enrollments and pregnancy dropouts comes from the Ministry of Education. Administrative data on baseline performance on the test scores comes from the Botswana Examinations Council. Standard deviations are in parentheses. Balance tests are conducted at the school level which was the unit of randomization. Analogous balance tests were conducted at the individual level for survey data with very similar results. The category "highest male HIV risk group" refers to identifying the 40-year old category as the correct one for being the highest HIV risk group.

Appendix B: At-Home Verification of Pregnancy

We verified pregnancies identified at the school by conducting at-home visits. This occurred from September through December 2015. Out of school surveys were conducted with both girls and their caregivers.* Girls received a written survey and an oral interview. This survey consisted of 35 questions nearly identical to the in-school endline survey. The written and oral survey were identical and the written survey was always taken first. Caregivers were interviewed orally. The caregiver survey contained 27 questions and contained similar questions on knowledge, attitudes and reported sexual behaviors about the girl. The surveys were conducted simultaneously with a physical structure or significant distance separating girls and their caregivers for privacy. If girls and caregivers were not able to be interviewed at the same time the girl was interviewed first.

A roll call of over 42,000 students was taken at the school and all girls who were missing due to pregnancy, dropout, or absence were followed up with a household visit.† In total, 588 girls were identified for at-home visits with a follow-up success rate of 99 percent of girls identified at the school as most likely to be pregnant were found. These follow-up visits required enumerators to survey the entire country, including meetings with local chiefs as well as to conduct visits across country borders in Zimbabwe and South Africa. Once at the household, enumerators observed whether the girl had a visibly large stomach (a 'tummy') or a baby. This was recorded. The enumerator would also give the girl a survey to fill out in written form followed by an oral interview. Finally, the enumerator would interview the girl's caregiver. In total this produced five measures of verification for pregnancy that had been identified at the school by classmates and teachers. Of all pregnancies identified at the school roughly 90 percent had at least one measure of the five indicate the girl was pregnant. This intensive follow up gives us confidence that our pregnancy measure is accurate and robust.

We compute the Cronbach's alpha to assess reliability of our various measures of pregnancy. We code tummy and baby as a joint observational measure. Across all measures, including written, oral and observational by the caregiver, girl and enumerator, we find a Cronbach's alpha of .65, which falls in the .60 to .70 range considered reliable in the measurement literature.

Item	Sign	Item-test Correlation	Alpha
Observational	+	.619	.638
Caregiver	+	.657	.598
Written (Girl)	+	.730	.598
Oral (Girl)	+	.790	.492
Chronbach's Alpha			.654

Notes: We collected 5 measures of pregnancy during household visits. This includes: caregiver surveys, written surveys with girls directly, oral surveys with girls directly, and measures of visible stomachs or 'tummies' and whether a baby was visible at home. While visible 'tummies' has not been validated in the literature, it provides a potentially objective measure of pregnancy to complement observations of babies. However, there are multiple interpretations of how tummy and baby measures move together. For example, tummies might indicate early pregnancy whereas babies indicate post-pregnancy. Given the ambiguities of how to interpret each measure independently we combine them into an observational measure which we corroborate against our three additional measures: interviews with girls and caregivers and survey responses from girls. Each row indicates the overall alpha if an item is removed, indicating its relative consistency.

* Of the 539 at-home visits, 504 included both a girl and caregiver out-of-school survey or the girl survey only when found in school, 22 included a caregiver survey only, 6 only include a girl survey only, and 7 girls were confirmed to be in school.

† A random sub-sample of girls who had transferred to schools outside of the study sample were also followed up to in case 'transfer' was a euphemism used by school classmates and administrators to cover up dropouts due to pregnancies.

Appendix C: Additional Results and Messenger Mechanism Exploration

A. Conceptual Framework for Messenger Mechanisms. The importance of messengers has received growing attention across psychology, public health, and economics. We explore messenger mechanisms along three primary channels: compliance, beliefs, and factors beyond beliefs. This conceptual framework highlights a series of possible messenger effects, the theoretical ambiguity of effects across different messengers, and the need for empirical evidence.

Compliance. Delivery of information will depend on showing up at school and transferring information undistorted. Although sex education is increasingly prioritized in government policies around the world, fewer than 35 percent of students across 79 countries have knowledge about basic HIV facts, and large gaps exist between teacher and student knowledge (1, 2). This reveals the importance of the compliance margin, suggesting that teachers might not deliver sex education as intended in the status quo. This might be since up to a fifth of teachers are absent in many developing countries (3, 4). Teachers might also be less comfortable delivering sensitive information that is considered taboo (5). In addition, since the information being sent reveals HIV risks by age, with older messengers revealed to be higher risk, the cost to older messengers and incentive to reveal information (about themselves) might be high. While multiple barriers exist for teachers to comply, it is not clear that near-peers will be more likely to comply. For example, near-peers are not always placed in schools full-time to begin with, introducing marginal costs to deliver sex education. Moreover, near-peers, who have zero years of teaching experience, might struggle to deliver the information as intended.

Beliefs. A literature in psychology explores the role of "source credibility" which posits that different sources of information can affect the likelihood that a message is perceived to be credible (6). Source credibility played an influential role during World War II, when the US government sought to use propaganda to influence public opinion in support of the war effort, and has received significant attention in psychology, marketing, communication and law. In the sex education context, teachers are often respected authority figures, and might be the most trustworthy sources of information. Connectedness might also matter. Banerjee et al. (7) find that "gossips" who are well connected in the community are most effective for in-network diffusion of information and in turn increasing rates of vaccination. Teachers are often well connected since they are based in the village of the receiver of information. On the other hand, near-peers, who are aspirational figures from the capital city, might be perceived to be more credible.

Factors beyond beliefs. Conditional on beliefs, messengers might be differentially persuasive. A teenager can listen, ignore, or rebel to information. Listening to a prescription derives from beliefs and can be enhanced through role model effects (8–10). The alternative option of rebellion has been explored extensively in psychology and described as "reactance." (11–14). We explore reactance through an economic lens, introducing agency into a teenager's utility function. A formalization of this notion is included later in the Supplement. Agency can be defined as a feeling that one is in control of their actions. Agency is threatened when a prescription is given by a person with authority, such as a teacher. Adhering to the prescribed action demonstrates lack of agency, since one "has to," resulting in disutility. Ignoring a prescription demonstrates partial agency, and performing the opposite action demonstrates full agency. Rebellion will occur if more utility is derived from exerting agency by ignoring the information or doing the exact opposite of the given prescription, even if listening to the prescription will yield economic payoffs. When a parent tells their child not to drink or smoke, they might now be motivated to try. When a teacher tells you to do your homework "or else" you might intentionally skip it. In these instances, there are clear economic payoffs to listening to the given advice, yet teenagers might forgo them and do the exact opposite to exercise maximum agency.

This framework delineates possible channels along the compliance, beliefs, and factors beyond beliefs margins through which the messenger can affect beliefs and behavior. This paper presents empirical evidence to shed light on which messenger mechanisms might be at play in our setting.

B. Results by Channel: Compliance, Beliefs, or Factors Beyond Beliefs. We conduct exploratory analysis by messenger mechanism channel. We start by examining results conditional on whether near-peers and teachers implement. For near-peers there is full implementation. However, for teachers implementation is partial. Implementation among teachers is captured using three core measures: attendance at training, commitment card signing, and returning of students' responses post intervention. All measures are imperfect but plausibly capture upper and lower bounds of implementation.

For our first analysis, we focus on implementation captured using the most conservative lower bound metric: data from student written responses sent by teachers to the regional ministry headquarters upon completion of the intervention. Around a third of all randomly selected teachers handed in this evidence of implementation. This is likely a lower bound of teacher implementation since teachers might implement but forget to send evidence of implementation or mail might get lost in transit. Quality checks were conducted. Open response answers were coded from students' reflections along four dimensions: wording, context, handwriting, and ink color. Uniqueness among these categories is coded as a proxy for the likelihood that these student reflections capture true implementation rather than fake data being filled out by the teacher. On average, just 0.1 percent appear to be fake responses. The open response format was designed to incentivize implementation since filling out hundreds of open responses by teachers is time-consuming, potentially even more so than implementing the one-hour intervention. The data indicates this was true in practice and that this measure reliably captures implementation by teachers.

For the sub-set of teachers that hand in evidence of implementation, beliefs update similarly to the near-peer arm. This suggests that conditional on implementation teachers can update beliefs as effectively as near-peers. Supplement Figure S6

depicts these results graphically showing convergence between near-peer and teachers for the subset that implement for HIV knowledge transfer.

Those who implement might be potentially selected, however we find in Supplement Table S3 that those who implement represent the broader population of teachers. Supplement Table S3 explores possible selection along baseline school characteristics, including test scores, dropouts and pregnancy, as well as teacher characteristics such as age, gender, experience, qualification and attitude towards the intervention. We run separate regressions with the independent variable coded to a dummy for whether teachers were randomly assigned as well as three measures of whether teachers implemented: attendance at training (90 percent of teachers), signing of commitment cards to deliver the intervention (74 percent of teachers), and returning of students' responses post intervention (30 percent of teachers). Results of each regression are reported in separate rows.

As shown, there appears to be no selection for the sub-sample of schools where teachers implement along any measure. While this is surprising at first glance, it is consistent with the context and design of the intervention. The intervention is only one hour. Thus, the marginal cost of delivering the intervention is very low. Moreover, schools already have time in the school scheduled to deliver sex education, again reducing barriers to entry and cost to delivery. Finally, all teachers randomly selected for training were guidance and counselling teachers and are thus already selected to engage with sex education at the outset. Most selection is likely between guidance and counselling teachers and other types of teachers, rather than within guidance and counselling teachers. With this context in mind, it is not entirely surprising that implementation was simply sporadic rather than selected. The lack of selection suggests that our teacher results are plausibly reflective of guidance and counselling teacher effectiveness in general beyond the sub-sample who implement.

Altogether, combining results from the main text figures and tables as well as the figures shown here in the Supplement, it appears that while teachers are less likely to comply and deliver the intervention in the first place, when they do, student beliefs update similarly to when near-peers deliver the intervention, suggesting that teachers and near-peers can be similarly effective messengers to transfer information.

We further conduct a formal treated-on-the-treated analysis using random assignment to exogenously instrument for implementation in Supplement Table S4 and estimate unbiased local average treatment effects. The first stage of each measure of implementation is highly significant as expected given they are a direct function of random assignment with t-statistics over 100 for each measure.

We estimate the effects of implementation and outcomes Y_{ij} as follows:

$$Y_{ij} = \alpha + \beta_1 I_j + \beta_2 I_j * teacher_j + \gamma X_j + \delta_s + \epsilon_{ij} \quad [1]$$

where I is one of our measures of implementation. Since implementation may be endogenous to teacher motivation and related variables we instrument for I using random assignment to receive the sex education message. We use a two-stage-least-squares (2SLS) estimation leveraging random assignment to instrument for implementation and then recover unbiased local average treatment effects of implementation on outcomes. These estimates are plausibly relevant to all teachers in the sample, given we observe limited selection of teachers who implement as shown in Supplement Table S3.

Supplement Table S4 shows instrumental variables (IV) estimates. Results demonstrate that the more likely teachers were to implement, the larger and more statistically significant the effects. The relative effect on pregnancy of teachers implementing the program increases from 0.253 significant at the 10 percent level (p-value = 0.051) to 0.426 significant at the 5 percent level (p-value = 0.026). This pattern is consistent for junior and rural girls. This indicates that teacher effects are unlikely to be a null driven by weak or non-implementation, but rather small increases in risky behavior. Of note, while larger effects might be a mechanical function of a smaller first stage, increased statistical significance points to an underlying mechanism driving these messenger effects.

Supplement Table S4 also shows corresponding results on beliefs by degree of implementation. The more likely teachers are to follow the expected intervention procedure, the more beliefs converge with near-peers, and in some cases teachers are able to update student beliefs even more than near-peers. Teachers who attend training converge to near-peers with approximately half the belief transfer as near-peers; teacher who commit to implementing have further convergence with near-peers with only a third less belief updating; teachers most likely to implement, evidenced by student responses, achieve full convergence, with no difference between near-peers and are in fact more effective at updating beliefs, especially in junior schools. Thus, results shows that beliefs converge between near-peers and teachers the more likely teachers are to implement. At the same time, the more likely teachers are to implement, and therefore the more beliefs update, the more pregnancy outcomes diverge. This suggests additional factors beyond beliefs are also likely to be playing a role in driving diverging behavioral responses. We conduct a corresponding mediation analysis. We find that beliefs seem to explain around 15 percent of the variation in treatment effects. This result is consistent with the notion that factors beyond beliefs are important in this setting.

C. The Teenager's Dilemma: Listen, Ignore, or Rebel. Students might listen, ignore, or rebel to the sex education message. If students listen, they will engage in safer sex. If students ignore or rebel, they might ignore the message or intentionally engage in riskier sex. Listening to a prescription derives straightforwardly from beliefs. In contrast, the option of rebellion is harder to explain. The possibility of teenage rebellion has been documented extensively in the psychology and sociology literature (11–14). This insight has spawned a technique used by the adept parent: reverse psychology. Anticipating rebellion, parents sometimes send the opposite message, such that when rebellion occurs, they get the desired response. Another parental favorite is soliciting a friend to give the desired prescription in the hopes their children will listen to someone else. In both cases, the messenger matters.

In the context of economics, we explore a potential explanation for rebellious behavior by including a sense of agency in a teenager's utility function, in a variation of the identity economics framework (15). Agency is defined as the capacity to act independently and make one's own choices. Agency is threatened when a prescription is given by a person with authority. Adhering to the prescribed action demonstrates lack of agency, since one "has to", resulting in disutility. Agency can be demonstrated by ignoring the prescription, demonstrating partial agency[‡], or performing the opposite action, demonstrating full agency, both of which enhance utility. Rebellion will occur if more utility is derived from exerting agency by doing the exact opposite of the given prescription, even if the given prescription will make you better off in terms of standard economic payoffs.[§]

When a parent tells their child not to drink or smoke, they might now be motivated to try. When a teacher tells you to do your homework "or else", you might intentionally skip it. In all these instances, there are clear economic payoffs to listening to the given advice, yet teenagers might forgo them and do the exact opposite to exercise maximum agency. This simple conceptual framework illuminates the possibility of rebellion. The results in this paper provide suggestive evidence such a response might be triggered depending on the messenger delivering a given message.

A Simple Utility Function Incorporating Agency. This section explores a utility function that incorporates agency as a potential motivator of behavior. Agency is defined as the capacity to act independently and make one's own choices. This builds on the concept of agency popularized in psychology (14) and a simple utility function in economics (15) which incorporates identity and self-image as a component of utility. This formulation further relates to an application in a school setting (16) which considers different types of students, such as "burnouts", who exhibit rebellious behavior. Rather than consider different types of students, we consider the same student with different potential behaviors.

We consider a utility function based on the actions one can take a_i and agency, A_i:

$$U_i = U_i(a_i, A_i)$$

where a_i determines consumption of goods and services, thus capturing the standard economic framework, and A_i can be represented by the following:

$$A_i = A_i(a_i, P_{ij}, G_{ij})$$

where a person i's agency depends on P_{ij}, a prescription given to a receiver i by a sender of information j, G_{ij}, a gradient of authority sender j has over receiver i, and how closely actions of person i, a_i, match the prescription P_{ij} given by person j to person i.

Agency can be threatened when a prescription is given by sender j with authority over receiver i, such as a teacher. In this case, adhering to the prescribed action demonstrates lack of agency, since one "has to." Performing no action demonstrates partial agency and performing the opposite action to the one corresponding to the prescription demonstrates full agency. When there is no prescription to rebel against, there is no opportunity to demonstrate extreme free will.

Consider a student making a choice on whether to take an action a in line with prescription P such that $a_i^P \in \{-1, 0, 1\}$ where $a_i^P = 1$ if they take the action as prescribed; $a_i^P = 0$ if they take no action; and $a_i^P = -1$ if they take the opposite action. The economic return to taking the action in line with the prescription is w. The effort involved in taking an action is denoted as e_i and the cost of effort $1/2e_i^2$. Let maximum agency $A = 1$, which we assume is the baseline case. Agency payoffs are the distance between maximum agency and the degree to which the action corresponds to the prescription weighted by the gradient of authority of the sender over the receiver G_{ij} such that $0 \leq G_{ij} \leq 1$. Together, we have a utility function for student i as follows:

$$U_i = \overbrace{(w * a_i^P)}^{economicpayoffs} + \overbrace{(1 - G_{ij} * a_i^P)}^{agencypayoffs} - 1/2e_i^2$$

In terms of economic payoffs, in the case of the sex education message and messengers in our study, if the prescription is acted on ($a_i^P = 1$) the student will have less unprotected sex, fewer teenage pregnancies, and related economic payoffs w,

‡ A possible outcome is that teachers have a null effect on pregnancy rather than increase it. This might be explained by students demonstrating 'partial agency' rather than 'full agency.' The near-peer arm demonstrates that in this particular context and for this particular sex education message belief updating *does* lead to changes in sexual behavior. Thus, it is unlikely environmental factors, such as financial constraints, are preventing information from translating to sexual behavior outcomes. Instead, a null could be driven by students demonstrating partial agency by ignoring teacher advice, rather than demonstrating full agency by rebelling against it.

§ An interesting question to explore is how much of the rebellion effect might be demonstrating agency for social image versus self-image. While we do not have direct evidence to answer this question, we hypothesize that in our context, social image is likely to be less tractable since rebellious actions taken are not directly and clearly observed. Whereas an action like not getting good grades is visible and often publicly shared in school settings, risky sexual encounters often occur in private, are taboo and rarely discussed publicly.

such as avoiding the cost of childrearing, or staying in school and having enhanced earning potential. If they take no action, they will be no better off economically such that the economic payoffs are 0. If they take the opposite action, they will incur negative economic payoffs $-w$, such as the cost of childrearing.

In terms of agency payoffs, if the sender has high authority over the student ($G_{ij} = 1$) and the action is taken according to the prescription ($a_i^P = 1$), agency payoffs are 0; if the sender has high authority over the student ($G_{ij} = 1$) and the action is not taken ($a_i^P = 0$), agency payoffs are 1; if the sender has high authority over the student ($G_{ij} = 1$) and the opposite action is taken ($a_i^P = -1$), agency payoffs are 2. This demonstrates the agency returns to ignoring, and especially rebelling, against a given prescription delivered by a sender with authority. When the agency payoffs to deviating from the prescription are higher than the economic payoffs of listening to the prescription, rebellion could occur.

If the sender has no authority over the student ($G_{ij} = 0$), all actions will yield agency payoffs of 1. In this case, actions have little agency returns, but have different economic payoffs and positive actions in line with the prescription will dominate. This demonstrates how students are inclined to listen to useful advice when their agency is not threatened.

This simple utility function serves to illustrate the potential for either a listening or rebellion response to the same set of information, depending on the sender of information, or messenger.

D. Additional Results. This section includes evidence of a listening versus rebellion response depending on the messenger by examining various dimensions of risky sexual behavior to supplement the main text results.

Additional outcomes. Supplement Table S5 shows effects on a series of self-reported sexual behaviors: number of sexual partners, if students have ever had sex in general and with older partners, if students are receiving gifts from a partner, if students feel a sense of self efficacy to say no to older partners, and condom use. Panels A through C focus on girls to most directly relate reported sexual behavior outcomes to earlier analyzed objective pregnancy outcomes, and where sexual behavior changes are most expected. In the near-peer arm girls exhibit greater empowerment and safer sexual behavior. Among girls in junior school this includes greater self-efficacy by 3.3 percentage points (p-value = 0.001) and girls being 2.2 percentage points (p-value = 0.028) less likely to have one or more sexual partner in junior school, an effect equivalent to an 11.5 percent reduction relative to the control average. In addition, we find junior school girls are 1.5 percentage points (p-value = 0.062) less likely to have had sex in the last 12 months, an effect equivalent to a 10 percent reduction. Moreover, rural girls in the near-peer arm are nearly a percentage point (p-value = 0.020) less likely to date older partners, an effect equivalent to a 15 percent reduction.[¶] In contrast, the relative effect of teachers is often in the opposite direction as near-peers, with high statistical significance in a few cases. Teachers increase the likelihood of receiving gifts from older partners relative to near-peers by 1.4 percentage points (p-value = 0.074). While results for near-peers alone are not significant, since results diverge between near-peers and teachers, the relative effect is significant. A similar pattern is observed for the likelihood of ever having sex for rural girls. Panels D and E show results for all students including boys, which show similar patterns. Overall effects are slightly smaller since behavioral changes for boys are expected to be marginal relative to girls. We interpret these results with caution given that self-reported behavior is susceptible to social desirability bias. However, these results support the notion that students listen to near-peers and engage in safer sexual behavior and might ignore or rebel against teachers.

Corresponding effects among boys and girls. In addition to data for girls, data for classmate boys might elucidate mechanisms. If students listen to the sex education message, they will follow the specific safer sex strategy proposed: dating younger partners. While we do not have reliable data on partner age or observe older partner behavior, we observe behavior among school-going boys. Since boys do not fall pregnant, we use the next best behavioral outcome available: attendance at school. We assume that both spending time with and matching with girls is one of young boys' incentives to be present in school. Thus, we expect boys to mimic girls' school-going behavior. If girls are in school since they are dating age-mates and haven't fallen pregnant, boys are more likely to be in school. In contrast, if girls shift to date older partners, they are more likely to spend time out of school. In turn, their male peers are less likely to come to school since there is no one to spend time or match with. In line with this prediction, we explore absenteeism among boys as a plausible corresponding behavioral response to pregnancy.

Supplement Table S6 shows results. Results are not statistically significant in the near-peer or teacher arm on their own. However, since effects go in opposite directions the relative effect of the near-peer versus teacher intervention is significant with 2 percentage points (p-value = 0.065) less absenteeism for boys in junior schools, translating into a 25 percent reduction. This behavioral response among boys is consistent with girls shifting to date lower-risk younger male partners whereas students rebel against teachers and shift towards dating higher-risk older partners.

Quantile treatment effects. Distributional effects are shown in Supplement Figure S7 using quantile treatment effect estimation. Effects along the distribution correspond to average treatment effects, with teacher and near-peer effects diverging at all points on the distribution. Effects are largest and most statistically significant at the upper end of the distribution.

[¶] Of note, we observe no changes in condom usage. This suggests that girls in the near-peer arm are more likely to delay sexual debut and not date at all or delay sexual debut when dating same-age partners, rather than negotiating for condom use.

E. Alternative Mechanisms.

Distortion of the message. Messengers might deliver the message but distort it. One possibility is that teachers revert to status-quo abstinence messaging. If this were the case, however, this would not drive differences relative to the control group, where the message is typically abstinence. Moreover, earlier results provide evidence that even a year later, students in the teacher arm learn that older partners are riskier and update their beliefs along the intensive margin, learning that older partners are riskier than younger partners. To this end, beliefs across the near-peer and teacher arms update in a similar direction and to a similar degree.

A related possibility is that teachers deliver a skewed message, emphasizing the risk of the *oldest* men and de-emphasizing the risk of *slightly older* men, thus inadvertently triggering entry into high-risk relationships with slightly older men. This possibility is captured in a conceptual framework where revision of beliefs downwards might trigger risky behavior in certain instances (17). For example, teachers might update HIV risk perceptions towards 40-year-olds away from 20-year-olds making 20-year-olds appear safer than students initially thought. In contrast, near-peers might update risk perceptions of all older partners, guarding against all high-risk partners. This pattern of belief updating across arms might explain why near-peers reduce pregnancy whereas teachers increase it. We find this explanation is unlikely to explain differences between teachers and near-peers in our context. Supplement Figure S8 shows that near-peers revise beliefs of 40-year-olds *upwards* relative to teachers and revise risk perceptions of 20-year-olds *downwards* relative to teachers. Thus, near-peers are more likely than teachers to de-emphasize the risk of slightly older men, not teachers, yet still reduce pregnancy.

Proximity. It is possible that students are most likely to act on beliefs when transferred by proximate messengers. Near-peers are more proximate than teachers to students on a bundle of characteristics such as age. Thus, as teachers converge towards near-peers in age, effects between near-peers and teachers might converge. Supplement Table S7 provides evidence of effects on pregnancy by degree of teacher proximity to students. Since teacher characteristics might be endogenous, we interpret these results as descriptive. The results suggest no relative effect of age and gender on pregnancy, possibly since teachers are authority figures regardless of their age. However, for each additional year of experience a teacher has, effects on pregnancy increase. This relationship holds for girls in junior school. This indicates that proximate teachers are no more effective, while more entrenched teachers – who often have more power and authority – drive pregnancy in the opposite direction. This suggests that authority over students might generate a rebellion response.

F. Mechanisms across contexts.. It is interesting that we find first-order effects of the messenger whereas a study in Cameroon found limited effects by messenger for a set of sex education messages (18). It is challenging to adjudicate exact reasons for this difference. Supplement Table S8 compares Kenya and Botswana, as well as a related study in Cameroon, for multiple indicators at the national level and for each study sample. The contexts in Botswana, Cameroon, and Kenya differ substantially, with up to a five times difference across dimensions such as income per capita, HIV rates, and sexual activity, so we are careful not to overstate drivers of variation without more conclusive evidence.

One possibility is that teachers are more trusted messengers in Cameroon. According to Demographic Health Survey (DHS) data condom knowledge is high in Cameroon at 82 percent relative to 65 in Botswana. However, comprehensive HIV knowledge is higher in Botswana at 48 percent relative to 31 percent in Cameroon, suggesting the direction of this effect is ambiguous. We further explore data from the Wellcome Trust Global Monitor Data in 2018 which explicitly measures levels of trust. Among young people aged 15-29 in Botswana, 57 percent trust science whereas 69 percent do in Cameroon. In contrast, slightly more youth in Botswana trust external sources such as NGOs than in Cameroon (54 relative to 47 percent). Since factual information is relatively less trusted in Botswana, but certain social actors such as NGOs are trusted more, this suggests the interaction between the type of information and its source might matter more in Botswana.

It is most likely that specific messenger profiles differ and matter. In Cameroon, for example, many teachers are contract teachers. Data from PASEC in 2011 reveal that 83 percent of primary school teachers in Cameroon are non-permanent (19). In Botswana less than 10 percent of teachers are non-permanent (20). These trends are consistent with higher rates of contract teachers in Francophone Africa than East and Southern Africa (19). Contract teachers are temporary, younger, and less formally trained. Thus, in practice contract teachers might converge in characteristics to external consultants resulting in fewer differences among messengers in Cameroon.

While challenging to reconcile differences across settings, our study in Botswana highlights that messengers can be first-order in important contexts with some of the highest HIV rates in the world. Moreover, the messengers studied in Botswana are of broad interest. 80 percent of teachers across 15 sub-Saharan African countries are estimated to be part of the civil service, rather than on contracts (21). Thus, estimates from this study provide consequential results on the most typical teachers delivering sex education at scale – full-time teacher civil servants – compared to a plausible alternative of near-peers.

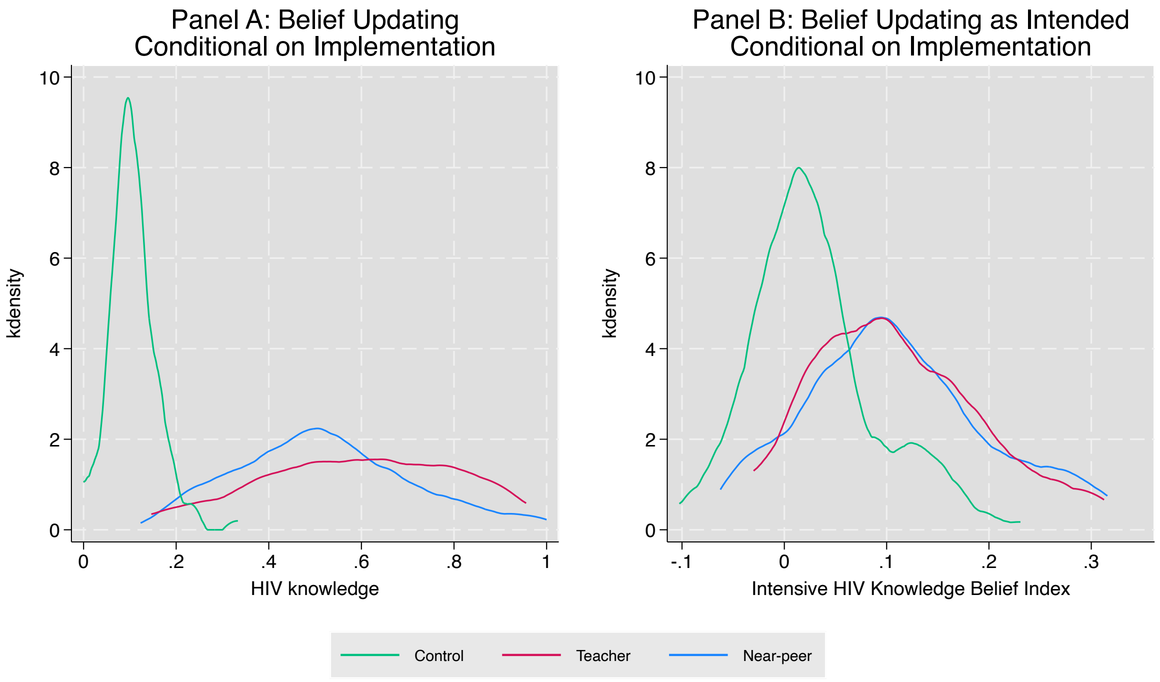

Fig. S6. Student Belief Updating Conditional on Implementation This figure presents belief updating by treatment arm conditional on implementation, defined as evidence of student responses submitted after the intervention. We show treatment effects on beliefs measured by HIV knowledge after the intervention. HIV knowledge is defined as knowing 40-year-old men are most likely to have HIV (among various age group categories students could select). We also present treatment effects on beliefs updating as intended (along the intensive margin). The index of beliefs captures the degree to which students think older partners have higher HIV than younger partners. The index is a linear subtraction of correctly identifying 40-year-old men as mostly to have HIV minus incorrectly identifying 10-19-year-old men as most likely to have HIV. Outliers are cutoff at 2.5 percentiles for clarity of visual exposition. Belief outcomes include all girls.

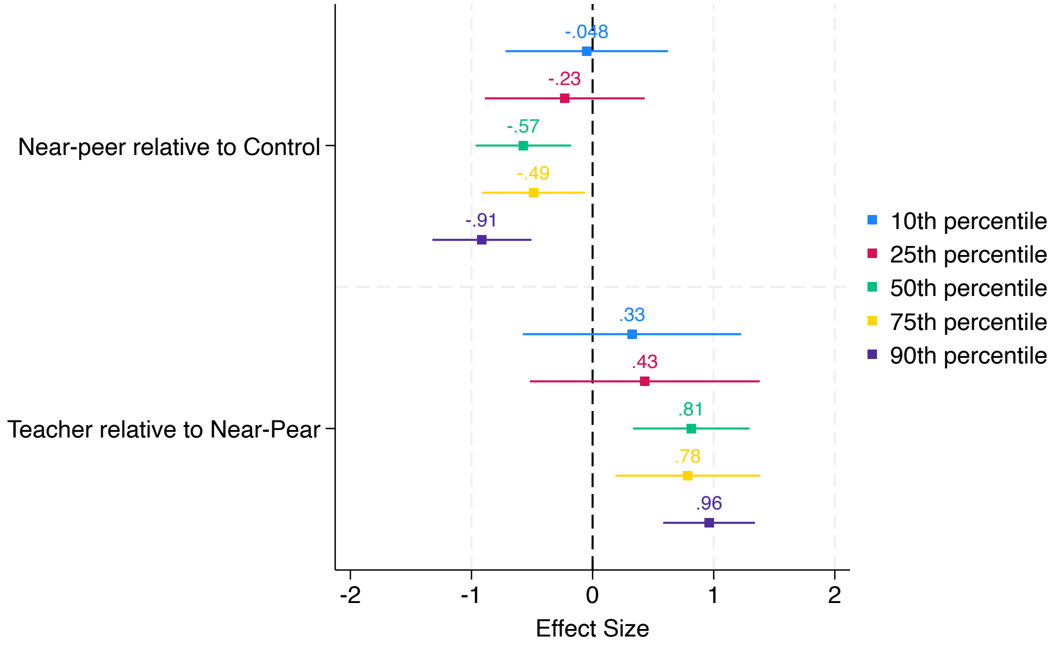

Fig. S7. Quantile Treatment Effects on Pregnancy Incidence Rates This figure plots quantile treatment effects on pregnancy annual incidence rates. The dependent variable is a school-level continuous variable for the percentage of girls pregnant. This variable construction differs slightly from individual-level pregnancy results with standard errors clustered at the school level. All specifications include strata dummies and a vector of school-level baseline control variables. We scale the dependent variable by a factor of one hundred for ease of interpretation of the coefficient as the direct incidence rate.

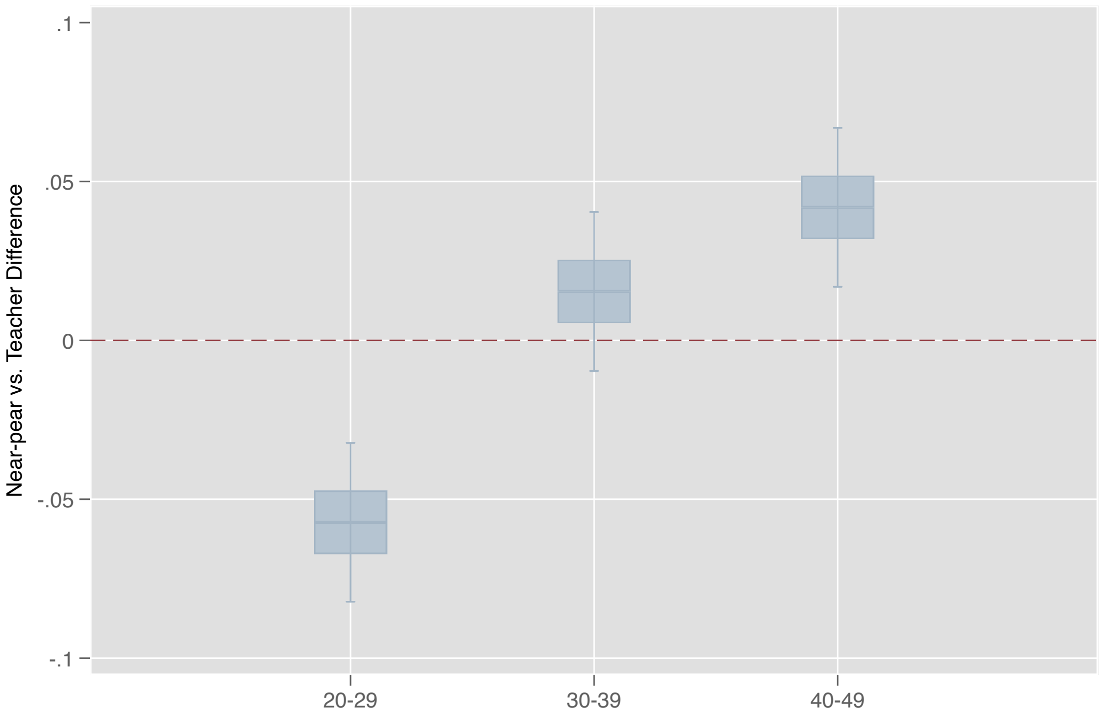

Fig. S8. HIV Risk Perception of Older Partners. This figure plots the relative difference in HIV risk belief updating between near-peers and teachers. This is a linear index of risk beliefs in the near-peer arm at endline relative to risk beliefs in the teacher arm for the question “which age group of men is most likely to have HIV.” We observe that near-peers update risk perceptions of 30-39-year-olds and 40-49-year-olds upwards relative to teachers and 20-29-year-olds downwards relative to teachers.

Table S3. Quantifying Selection for Teachers Who Implement

	Baseline School Characteristics				Teacher Characteristics				
	(1)	(2)	(3)	(4)	(5)	(6)	(7)	(8)	(9)
	Female	Test Scores	Pregnancy	Dropout	Age	Gender	Experience	Bachelors	Diploma
Random Assignment	-0.001	0.501	-0.108	-0.158	0.819	0.012	0.195	-0.001	-0.023
	(0.011)	(0.774)	(0.118)	(0.409)	(0.636)	(0.047)	(0.813)	(0.038)	(0.054)
	[0.927]	[0.518]	[0.360]	[0.700]	[0.198]	[0.805]	[0.810]	[0.982]	[0.673]
Attended Training	0.003	0.839	-0.117	-0.008	0.870	-0.021	0.468	0.013	-0.016
	(0.012)	(0.728)	(0.105)	(0.420)	(0.667)	(0.048)	(0.831)	(0.040)	(0.056)
	[0.809]	[0.250]	[0.268]	[0.986]	[0.193]	[0.660]	[0.574]	[0.742]	[0.778]
Committment Card	-0.006	0.506	-0.062	0.148	0.328	0.010	0.034	-0.005	-0.027
	(0.013)	(0.794)	(0.109)	(0.487)	(0.729)	(0.049)	(0.866)	(0.041)	(0.059)
	[0.631]	[0.524]	[0.570]	[0.761]	[0.654]	[0.844]	[0.969]	[0.894]	[0.649]
Student Responses	0.019	0.033	-0.083	0.385	0.558	0.061	-0.361	-0.043	-0.104
	(0.018)	(0.963)	(0.110)	(0.540)	(0.886)	(0.059)	(0.995)	(0.043)	(0.085)
	[0.288]	[0.972]	[0.447]	[0.476]	[0.529]	[0.302]	[0.716]	[0.309]	[0.226]
Control Mean	0.512	52.783	0.452	2.096	43.465	0.737	21.138	0.096	0.623
Observations	339	339	343	343	340	337	336	339	339

This table compares the teacher arm to the control arm and reports effects of teacher selection by degree of implementation using baseline average school characteristics as well as teacher characteristics. We run a separate regression per row, running a regression coded to a dummy of all randomly assigned teachers as well as three measures of whether teachers implemented: attendance at training (90 percent of teachers), signing of commitment cards to deliver the intervention (74 percent of teachers), and returning of students' responses post intervention (30 percent of teachers). Baseline data on dropouts and pregnancy are from the Ministry of Education and baseline test scores are from the Botswana Education Council. Teacher characteristics data comes from endline surveys of guidance and counselling school teachers across all arms. We code non-implementation by each measure of implementation as a zero. We see no evidence of selection across all indicators by any degree of implementation. All specifications include strata dummies. Each row is a separate regression, comparing teachers in general and teachers who implement to the near-peer and control groups to identify potential selection effects. Standard errors are in parentheses and p-values are in square brackets.

Table S4. Treatment on the Treated Effects on Pregnancy and Beliefs

	Pregnancy Incidence Rate			Beliefs Post Intervention		
	(1) All Girls	(2) Junior Girls	(3) Rural Girls	(4) All Girls	(5) Junior Girls	(6) Rural Girls
Panel A: Attended Training						
Sex Education	-0.244	-0.492	-0.383	0.436	0.452	0.429
	(0.090)	(0.132)	(0.105)	(0.019)	(0.033)	(0.022)
	[0.007]	[0.000]	[0.000]	[0.000]	[0.000]	[0.000]
Sex Education x Teacher	0.253	0.574	0.437	-0.215	-0.247	-0.172
	(0.130)	(0.215)	(0.147)	(0.043)	(0.067)	(0.052)
	[0.051]	[0.008]	[0.003]	[0.000]	[0.000]	[0.001]
Panel B: Comittment Card						
Sex Education	-0.230	-0.473	-0.354	0.416	0.425	0.412
	(0.095)	(0.131)	(0.112)	(0.021)	(0.034)	(0.024)
	[0.016]	[0.000]	[0.002]	[0.000]	[0.000]	[0.000]
Sex Education x Teacher	0.295	0.792	0.538	-0.158	-0.167	-0.104
	(0.138)	(0.308)	(0.140)	(0.055)	(0.102)	(0.056)
	[0.032]	[0.010]	[0.000]	[0.004]	[0.102]	[0.065]
Panel C: Student Responses						
Sex Education	-0.221	-0.478	-0.379	0.412	0.430	0.417
	(0.093)	(0.129)	(0.107)	(0.014)	(0.015)	(0.016)
	[0.017]	[0.000]	[0.000]	[0.000]	[0.000]	[0.000]
Sex Education x Teacher	0.426	1.049	0.524	0.098	0.195	0.094
	(0.191)	(0.384)	(0.196)	(0.038)	(0.046)	(0.040)
	[0.026]	[0.006]	[0.008]	[0.009]	[0.000]	[0.018]
Control Mean	0.494	0.737	0.566	0.100	0.099	0.102
Observations	22907	14753	17460	22491	14380	17233

This table reports reduced form instrumental variables (IV) estimates of treatment effects on pregnancy and beliefs by the degree of teachers complying with the expected procedure. Beliefs include students who identify older partners are most likely to have HIV shortly after the intervention. Implementation is coded as “Attended” if teachers attended training, “Commitment Card” if teachers signed a card scheduling implementation and “Student Responses” if teachers handed back written students’ responses to the regional ministry authority post implementation. The bounds on these measures of implementation range from 90 percent to 30 percent. Attended, Commitment Card and Student Responses are instrumented for by random assignment to the teacher arm. All specifications are 2SLS and include strata dummies and a vector of school-level baseline control variables. We scale the pregnancy dependent variable by a factor of one hundred for ease of intuitive interpretation of the coefficient as the direct incidence rate. All standard errors are robust and clustered at the school-level. Standard errors are in parentheses and p-values are in square brackets.

Table S5. Mechanisms – Self-Reported Sexual Behavior

	(1) > 1 Sex Partner	(2) Had Sex	(3) Sex w/ Older	(4) Given Gifts	(5) Self Efficacy	(6) Condom Use
Panel A: All Girls						
Sex Education	-0.014	-0.009	-0.005	-0.005	0.018	-0.010
	(0.009)	(0.006)	(0.003)	(0.005)	(0.012)	(0.006)
	[0.132]	[0.097]	[0.113]	[0.319]	[0.120]	[0.119]
Sex Education x Teacher	0.015	0.015	0.006	0.010	-0.008	0.007
	(0.011)	(0.007)	(0.004)	(0.007)	(0.012)	(0.008)
	[0.164]	[0.038]	[0.166]	[0.124]	[0.506]	[0.330]
Control Mean	0.110	0.074	0.030	0.067	0.805	0.065
Observations	21148	21114	20991	20944	21030	20981
Panel B: Junior Girls						
Sex Education	-0.022	-0.015	-0.003	-0.006	0.033	-0.013
	(0.010)	(0.008)	(0.005)	(0.008)	(0.009)	(0.008)
	[0.028]	[0.062]	[0.561]	[0.447]	[0.001]	[0.123]
Sex Education x Teacher	0.023	0.016	0.003	0.014	-0.027	0.009
	(0.011)	(0.008)	(0.005)	(0.008)	(0.011)	(0.009)
	[0.036]	[0.056]	[0.559]	[0.074]	[0.016]	[0.299]
Control Mean	0.119	0.096	0.032	0.082	0.836	0.082
Observations	13575	13545	13438	13399	13456	13424
Panel C: Rural Girls						
Sex Education	-0.018	-0.012	-0.008	-0.006	0.014	-0.012
	(0.012)	(0.007)	(0.003)	(0.007)	(0.014)	(0.008)
	[0.145]	[0.089]	[0.020]	[0.394]	[0.342]	[0.132]
Sex Education x Teacher	0.017	0.016	0.010	0.012	-0.009	0.010
	(0.014)	(0.009)	(0.005)	(0.009)	(0.015)	(0.010)
	[0.223]	[0.065]	[0.028]	[0.190]	[0.545]	[0.317]
Control Mean	0.125	0.079	0.033	0.073	0.783	0.073
Observations	16164	16142	16040	16012	16083	16040
Panel D: All Students						
Sex Education	-0.007	-0.003	-0.002	-0.004	0.001	0.001
	(0.010)	(0.006)	(0.003)	(0.005)	(0.012)	(0.006)
	[0.492]	[0.555]	[0.652]	[0.359]	[0.937]	[0.869]
Sex Education x Teacher	0.011	0.013	0.004	0.012	0.002	0.001
	(0.010)	(0.007)	(0.003)	(0.005)	(0.012)	(0.007)
	[0.281]	[0.051]	[0.270]	[0.034]	[0.842]	[0.858]
Control Mean	0.174	0.122	0.049	0.092	0.711	0.100
Observations	41377	41245	41086	40963	41144	41238
Panel E: All Junior Students						
Sex Education	-0.013	-0.003	0.003	-0.007	0.017	0.004
	(0.011)	(0.009)	(0.005)	(0.007)	(0.012)	(0.009)
	[0.234]	[0.749]	[0.583]	[0.327]	[0.178]	[0.662]
Sex Education x Teacher	0.022	0.014	0.001	0.019	-0.025	0.001
	(0.011)	(0.008)	(0.005)	(0.006)	(0.012)	(0.008)
	[0.051]	[0.059]	[0.798]	[0.001]	[0.049]	[0.874]
Control Mean	0.192	0.152	0.055	0.109	0.732	0.125
Observations	26220	26129	25953	25868	25970	26069

Notes: This table reports treatment effects on self-reported sexual behavior and attitudes for girls and boys after 12 months. All standard errors are robust and clustered at the school-level. All specifications include strata dummies and a vector of school-level baseline control variables. Standard errors are in parentheses and p-values are in square brackets.

Table S6. Mechanisms – Treatment Effects on Boys' Absenteeism

	Absenteeism Incidence		
	(1)	(2)	(3)
	All Boys	Junior Boys	Rural Boys
Sex Education	-0.416	-0.759	-0.800
	(0.766)	(0.969)	(0.899)
	[0.587]	[0.436]	[0.374]
Sex Education x Teacher	0.880	2.038	0.939
	(0.732)	(1.085)	(0.803)
	[0.231]	[0.065]	[0.243]
Control Mean	8.077	8.410	8.284
Observations	22288	13993	17159

Notes: This table reports treatment effects on boys' absenteeism from school. Boys' absenteeism is calculated using the 'roll-call' method where enumerators read off a list of all students' names from baseline to see who was missing from school at endline. All standard errors are robust and clustered at the school-level. All specifications include strata dummies and a vector of school-level baseline control variables. We scale the dependent variable by a factor of one hundred for ease of intuitive interpretation of the coefficient as the direct incidence rate. Standard errors are in parentheses and p-values are in square brackets.

Table S7. Mechanisms – Effects on Pregnancy by Teacher Proximity

	Pregnancy Incidence Rate				
	(1)	(2)	(3)	(4)	(5)
Teacher	-1.682	0.099	-1.652	-1.804	-1.001
	(2.089)	(0.201)	(2.143)	(0.429)	(0.525)
	[0.425]	[0.625]	[0.445]	[0.000]	[0.063]
Age	-0.658		-0.643		-0.432
	(0.199)		(0.218)		(0.116)
	[0.002]		[0.005]		[0.001]
Teacher x Age	0.294		0.288		
	(0.377)		(0.389)		
	[0.439]		[0.463]		
Gender		0.100			
		(0.209)			
		[0.633]			
Experience			-0.004	-0.100	-0.046
			(0.011)	(0.015)	(0.024)
			[0.756]	[0.000]	[0.065]
Teacher x Experience				0.099	0.053
				(0.024)	(0.029)
				[0.000]	[0.075]
Observations	9304	9304	9304	9304	9304

Notes: This table reports treatment effects on pregnancy by teacher proximity to students on a bundle of characteristics including age (in years), gender and teaching experience (in years). We run a regression interacting the teacher treatment (relative to control) with the teacher's age and years of experience teaching. Age is coded as a continuous number for the teacher's age and experience is coded as a continuous variable for the years of experience the teacher has been teaching, both elicited from direct surveys with teachers. Since teacher characteristics might be endogenous, we interpret these results as descriptive and suggestive. We report effects at junior school. Standard errors are robust and clustered at the school-level. All specifications include strata dummies and a vector of school-level baseline control variables. We scale the dependent variable by a factor of one hundred for ease of intuitive interpretation of the coefficient as the direct percentage. Standard errors are in parentheses and p-values are in square brackets.

Table S8. Comparison Across Contexts

	(1) Kenya 2004	(2) Cameroon 2011	(3) Botswana 2014
Panel A: National Indicators			
GDP per capita (in current USD)	458.88	1403.23	7493.75
Percent of adults aged 15-49 living with HIV	0.07	0.04	0.23
Percent of all people living with HIV accessing ARVs	0.02	0.20	0.69
Total fertility rate (births per woman)	4.90	5.05	2.80
Gross primary enrollment ratio	1.02	1.07	1.05
Net primary enrollment rate	0.70	0.88	0.89
Population density (people per square kilometer)	61.60	44.22	3.80
Share of the population that lives in urban areas	0.21	0.52	0.66
Comprehensive HIV Knowledge	0.42	0.31	0.48
Share Non-permanent or Contract Teachers	0.24	0.83	0.10
Trust in Science	0.71	0.69	0.57
Trust in NGOs	0.66	0.47	0.54
Panel B: Study sample			
Age (years)	15.10	16.77	13.60
Class size	38.20	23.00	36.80
Share reporting having had sex	0.21	0.22	0.07
Sharing reporting having fallen pregnant	0.14	0.10	0.05
Share that think men $>$ 25 have higher HIV rates than teen boys	0.29	0.39	0.50
Share that think condoms can prevent HIV	0.45	0.82	0.65

Notes: We compare national-level indicators and study samples in Kenya, Cameroon, and Botswana along the most similar indicators available. For study-specific samples, we draw on numbers reported by Dupas (17) and Dupas, Huillery, and Seban (18) directly. Since some indicators are only reported along various dimensions of disaggregation such as gender and by treatment group, we report the most direct analogy from our study. Study indicators are reported for girls only while national-level indicators are reported for both genders. Class size is reported for the control groups in the Kenya and Botswana studies to ensure comparability and on average at baseline for Cameroon. The share of students that think men over 25 have higher rates of HIV than teenage boys is derived in this paper by calculating the percentage of students who say 30-year-old men and older are most likely to have HIV since the surveys used in this study grouped risk profiles by 10-year age buckets. While this is not the identical question reported in the other studies which used different age buckets (such as over $>$ 25) it is very similar and captures a comparable indicator of knowledge of HIV risks.

Source: National-level indicators are compiled by the World Bank drawing on estimates from UNAIDS, UNDP, UIS, FAO, World Bank national accounts data and the Demographic Health Surveys (DHS). Baseline from the study samples are drawn from Dupas (17) for Kenya, from Dupas, Huillery, and Seban (18) for Cameroon, and from the baseline in this study for Botswana. For the share of contract or non-permanent teachers, this data for Cameroon and Kenya comes from the nearest year available, 2011 and 2007, respectively from SACMEQ and PASEC surveys. For Botswana, this data come from a report by UNICEF from data in 2017. Trust data from the Wellcome Global Monitor is available in 2018 so we can compare this indicator across countries, but it is not specific to the years across trials.

Comparing Results. Here we describe how the final results presented in this paper relate to preliminary work mentioned in early blog posts by implementing and funding partners. As the analysis sharpened over time, the main story in the paper became clear: the messenger matters, with reductions in pregnancy when delivered by near-peers, and no reductions when delivered by teachers. An earlier post mentions an alternative measure of pregnancy called a 'tummy test' which was tried for this study, but had not been previously validated in the literature. For this test, enumerators observed large stomachs and recorded them. While the main validated measure of pregnancy used in the study decreased in the treatment group, this 'tummy' measure had initially increased in the treatment group. One plausible interpretation for this result would be a *delay* in pregnancy rather than a *prevention* in pregnancy. If fewer girls would have been pregnant 12 months before, as evidenced by the main measure of pregnancy used in the study, they would then be susceptible to falling pregnant shortly after, leading to early stage visible 'tummies' by the time of the endline survey. However, while initial discussion aimed to interpret the tummy test sensibly, various experts have since refuted this measure and recommended using it to triangulate other measures rather than as a stand-alone measure. A paper published in a public health journal which reports a simple set of results from only one treatment arm relative to a control group underwent peer review where expert guidance was provided on the most reliable pregnancy measure (22).[‖,**]

The complexity of the initial results led to an early interpretation that effects were ambiguous; over time, through peer review and careful data analysis, this interpretation was sharpened and focused: the messenger matters, with reductions in pregnancy when delivered by near-peers, and no reductions when delivered by teachers. A few additional lessons emerged which have since been incorporated into future programming.[††] The key policy implication for the program, which initially considered a rapid scale up with teachers, was to use lessons from the evidence to improve the program prior to scaling up, such as refining delivery via scalable near-peer models (e.g., national service programs).[‡‡]

[‖] We thank Esther Duflo, Pascaline Dupas, Rachel Glennerster, and Taveneet Suri for various helpful discussions on interpretation of this measure, as well as multiple public health experts including anonymous peer reviewers.

[**] Of note, this paper provides substantial new results over earlier work published in public health. Most crucially, this paper provides novel results comparing the results of two treatment groups which varied the messenger alongside a conceptual framework and novel suite of outcomes and estimation procedures to explore messenger mechanisms.

[††] The data suggest that older partners included all of the above: 20s, 30s, and 40s. The program which initially targeted the oldest partners in their 40s has since evolved to include messaging about the risk of partners in all older age categories including 20s, 30s, and 40s. An early blog post mentioned that effects on knowledge fade over time. As shown in the main text, knowledge gains reduce from over 30 percentage point gains after the intervention to around 10 percentage point gains a year later. To maximize persistence of knowledge, additional low-cost SMS and phone call booster messages have since been incorporated into the program. While knowledge fades, it is notable that some knowledge persists a full year later, with elements of a brief, low-cost intervention sustaining over time and the main knowledge indicator of interest likely remains initial knowledge transfer, since this is what results in lower pregnancy 9-12 months later.

[‡‡] Of note, this paper updates an earlier working paper version (23)

1. UNAIDS, Global hiv & aids statistics–2018 fact sheet, (Geneva, Switzerland), Technical report (2018).
2. UNESCO, Unesco's strategy for hiv and aids, (UNESCO Paris, France), Technical report (2011).
3. N Chaudhury, J Hammer, M Kremer, K Muralidharan, FH Rogers, Missing in action: teacher and health worker absence in developing countries. *J. Econ. Perspectives* **20**, 91–116 (2006).
4. E Duflo, R Hanna, SP Ryan, Incentives work: Getting teachers to come to school. *Am. Econ. Rev.* **102**, 1241–1278 (2012).
5. DA Ross, B Dick, J Ferguson, WH Organization, *Preventing HIV/AIDS in young people: a systematic review of the evidence from developing countries.* (World Health Organization), (2006).
6. CI Hovland, W Weiss, The influence of source credibility on communication effectiveness. *Public Opin. Q.* **15**, 635–650 (1951).
7. AV Banerjee, AG Chandrasekhar, E Duflo, MO Jackson, Using gossips to spread information: theory and evidence from two randomized controlled trials. *Rev. Econ. Stud.* (2019).
8. E La Ferrara, A Chong, S Duryea, Soap operas and fertility: Evidence from brazil. *Am. Econ. Journal: Appl. Econ.* **4**, 1–31 (2012).
9. T Bernard, S Dercon, K Orkin, AS Taffesse, Will video kill the radio star? assessing the potential of targeted exposure to role models through video. *The World Bank Econ. Rev.* **29**, S226–S237 (2015).
10. E La Ferrara, Mass media and social change: Can we use television to fight poverty? *J. Eur. Econ. Assoc.* **14**, 791–827 (2016).
11. JW Brehm, *A theory of psychological reactance.* (Academic Press, Oxford), (1996).
12. JW Pennebaker, DY Sanders, American graffiti: Effects of authority and reactance arousal. *Pers. Soc. Psychol. Bull.* pp. 264–267 (1976).
13. BJ Bushman, AD Stack, Forbidden fruit versus tainted fruit: Effects of warning labels on attraction to television violence. *J. Exp. Psychol. Appl.* **2**, 207 (1996).
14. A Bandura, Social cognitive theory: An agentic perspective. *Annu. Rev. Psychol.* **52**, 1–26 (2001).
15. GA Akerlof, RE Kranton, Economics and identity. *The Q. J. Econ.* **115**, 715–753 (2000).
16. GA Akerlof, RE Kranton, Identity and schooling: Some lessons for the economics of education. *J. Econ. Lit.* **40**, 1167–1201 (2002).
17. P Dupas, Do teenagers respond to hiv risk information? evidence from a field experiment in kenya. *Am. Econ. Journal: Appl. Econ.* **3**, 1–34 (2011).
18. P Dupas, E Huillery, J Seban, Risk information, risk salience, and adolescent sexual behavior: Experimental evidence from cameroon. *J. Econ. Behav. & Organ.* **145**, 151–175 (2018).
19. S Bashir, M Lockheed, E Ninan, JP Tan, *Facing forward: Schooling for learning in Africa.* (The World Bank), (2018).
20. UNICEF, Public expenditure review of the basic education sector in botswana, Technical report (2019).
21. DK Evans, F Yuan, D Filmer, Teacher pay in africa: Evidence from 15 countries. *World Dev.* **155**, 105893 (2022).
22. N Angrist, M Matshaba, L Gabaitiri, G Anabwani, Revealing a safer sex option to reduce hiv risk: a cluster-randomized trial in botswana. *BMC Public Heal.* **19**, 1–8 (2019).
23. N Angrist, Ph.D. thesis (University of Oxford) (2020).

327 **Survey Tool Sample**

1
[_|_|_]

In-school Anonymous Survey: Females

We are carrying out a study to try and improve health education for youth in Botswana. This is not a test, and no answers are wrong. We are interested to learn your opinions. Your participation in this survey is voluntary, and your answers will be used anonymously. They will not be shared with your family, friends, teachers, or anyone in my community. If you don't want to answer a question, you may choose to skip it. If you don't understand a question or would like it translated into Setswana, you can put up your hand and a surveyor will explain it to you privately.

#	QUESTION	ANSWER
1	Date	Day[__|__] Month[__|__] Year[__|__|__|__]
2	School Name	
3	School ID #	[__|__|__]
4	Surveyor ID #	[__|__|__]
5	Standard/Form	[__] Std. 7 [__] Form 2 [__] Form 3
6	Which Standard or Form were you in last year (in 2014)?	[__] Std. 6 [__] Std. 7<br>[__] Form 1 [__] Form 2 [__] Form 3
7	When were you born?	Day[__|__] Month[__|__] Year[__|__|__|__]
8	Are you a girl or a boy?	[__] Boy [__] Girl
9	Which do you consider most important in choosing a sexual partner?<br>(Pick only ONE)	[__] Wealth [__] Condom use<br>[__] HIV status [__] Faithfulness<br>[__] Social status [__] Other: _______________
10	Who do you think has the highest risk of infecting you with the HIV/AIDS virus?<br>(Pick only ONE)	[__] Your age mates<br>[__] Other young people<br>[__] Older people<br>[__] Don't know
11	When used correctly and consistently, do you think that condoms can prevent pregnancy?<br>(Pick only ONE)	[__] All the time (100%)<br>[__] More than half the time, but not always<br>[__] Less than half the time<br>[__] They do not prevent pregnancy<br>[__] Don't know
12	When used correctly and consistently, do you think that condoms can prevent HIV/AIDS?<br>(Pick only ONE)	[__] All the time (100%)<br>[__] More than half the time, but not always<br>[__] Less than half the time<br>[__] They do not prevent HIV/AIDS<br>[__] Don't know
13	The age group of men most likely to have HIV is:<br>(Pick only ONE)	[__] 10-19 [__] 40-49<br>[__] 20-29 [__] 50 and above<br>[__] 30-39
14	The age group of women most likely to have HIV is:<br>(Pick only ONE)	[__] 10-19 [__] 40-49<br>[__] 20-29 [__] 50 and above<br>[__] 30-39
15	As far as you know, about how many girls in your class have a boyfriend 5 or more years older than them?	[__|__]<br>[__] Don't know
16	Have you ever been tested for HIV or other sexually transmitted infections?	[__] Yes [__] No
17	Have you ever had sexual intercourse?<br>(Pick only ONE)	[__] Yes, with more than one person<br>[__] Yes, with only one person<br>[__] No
18	How many people have you had sex with in the last 12 months?<br>(Pick only ONE)	[__] None [__] 11-20<br>[__] 1-5 [__] 21 and above<br>[__] 6-10

F1

1
[__|__|__]

In-school Anonymous Survey: Females

19	Have you ever used a condom while having sex?<br>(Pick only ONE)	[__] Yes, every time I had sex<br>[__] Yes, some of the times I had sex<br>[__] Yes, once<br>[__] No<br>[__] Not applicable
20	Do you know how to use a condom correctly during sex?<br>(Pick only ONE)	[__] Yes, perfectly (100%)<br>[__] Yes, moderately<br>[__] Yes, a little<br>[__] Not at all<br>[__] I have never used a condom
21	Are you able to get condoms whenever you need them for sex?<br>(Pick only ONE)	[__] All the time (100%)<br>[__] More than half the time, but not always<br>[__] Less than half the time<br>[__] Not at all<br>[__] I have never tried to get a condom
22	Are you worried about the consequences of getting pregnant?	[__] Yes [__] No
23	Have you had sex with a person who is 5 or more years older than you in the last 12 months?	[__] Yes [__] No
24	Have you had a sexual partner in the last 12 months who helped **you** out financially/with gifts?	[__] Yes [__] No
25	Have you had a sexual partner in the last 12 months who helped out **your family** financially/with gifts?	[__] Yes [__] No
26	Who do you mostly depend on financially now?	[__] I take care of myself<br>[__] My parents<br>[__] My partner<br>[__] My child's father<br>[__] Another family member<br>[__] Other: ________________________
27	Have you ever been pregnant?	[__] Yes [__] No
28	If so, how far along is the pregnancy?	[__|__] months pregnant<br>[__|__] months old baby<br>[__] Don't know<br>[__] Not applicable
29	Are you still in a relationship with the man who got you pregnant?	[__] Yes [__] No<br>[__] Not applicable
30	How old was the man who got you pregnant?	[__|__] years<br>[__] Not applicable
31	Did you know the man's HIV status when he got you pregnant?	[__] Yes [__] No<br>[__] Not applicable
32	Did the man who got you pregnant have HIV?	[__] Yes [__] No<br>[__] Not applicable [__] Don't know
33	**Before** you fell pregnant, did the man who got you pregnant help you financially or give you gifts?	[__] Yes [__] No<br>[__] Not applicable
34	I think I could say no to sex with a partner 5 or more years older than me.<br>(Pick only ONE)	[__] Yes [__] No [__] Maybe

Thank you for taking your time to answer these questions.

F2
